# Catalyst‐Free Cyanation of Arenes by Acetonitrile in a Plasma–Liquid Microreactor

**DOI:** 10.1002/chem.71101

**Published:** 2026-05-13

**Authors:** Pierre Dedieu, Gabriel Morand, Stéphanie Ognier, Alain Favre‐Réguillon, Michael Tatoulian

**Affiliations:** ^1^ Institut de Recherche de Chimie Paris, UMR 8247 Chimie ParisTech ‐ PSL Paris France; ^2^ UFR Chimie Sorbonne Université Paris France; ^3^ EPN 7 ”Chimie, Santé Vivant” Conservatoire National des Arts et Métiers La Plaine Saint‐Denis France

**Keywords:** arylnitriles, C‐H activation, cyanation, microreactors, plasma chemistry

## Abstract

A catalyst‐free direct C‐H cyanation of arenes was performed in continuous flow by treating aromatic substrates in acetonitrile with an argon plasma in a gas–liquid microreactor under ambient conditions. The reactivity of the different radicals generated through plasma activation of acetonitrile was probed using benzene as a model substrate, producing benzonitrile in a 25% yield. Mechanistic insights derived from kinetic modeling and isotopic labeling experiments enabled the proposal of a reaction pathway accounting for the formation of the main nitrile products. Expanding the substrate scope demonstrated consistent nitrile formation aligned with the mechanistic hypothesis. This catalyst‐free direct C‐H cyanation of arenes using an accessible and low‐toxicity nitrile source highlights the promising role of plasma activation as an alternative tool for organic transformations.

## Graphical abstract

An original approach of arylnitrile synthesis was developed by generating an argon plasma in contact with solutions of arene in acetonitrile within a gas‐liquid microreactor. Plasma activation of acetonitrile generated cyano radicals, enabling a catalyst‐free C‐H cyanation of arenes. This work highlights the potential of plasmas to uncover new sustainable synthetic pathways in organic chemistry.

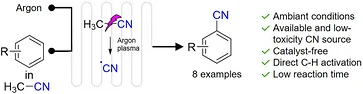

## 1 Introduction

The arylnitrile moiety is today recognized as a key structural motif throughout chemistry. The intrinsic properties of the nitrile group make it of particular interest in medicinal chemistry [1] and polymer science [2]. Nitriles are also versatile intermediates in organic chemistry that can be valued into multiple functional groups such as carbonyls, amines, imines, and N‐heterocyclic compounds like tetrazoles [3, 4, 5]. These features have contributed to the growing interest in synthesizing arylnitriles as valuable building blocks.

**FIGURE 1:**
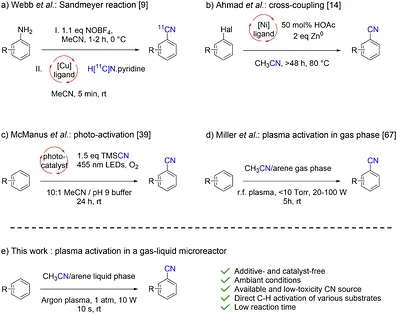
Classical arene cyanation reactions and the strategy used in this work (R: substituent, Hal: halogen atom).

Traditional arylnitrile syntheses include the Sandmeyer reaction starting from anilines (Figure 1a) [6, 7, 8] and cross‐coupling reactions using mainly arylhalides and metal‐based catalysts (Figure 1b) [9, 10, 11, 12, 13, 14, 15]. However, these traditional strategies still need to overcome several challenges. First, the predominant use of activated substrates limits the scope of cyanation reactions and induces additional synthesis steps. Although different direct C‐H cyanation reactions have been developed, they still usually require either directing groups on the substrate or the use of additives [16, 17, 18, 19, 20, 21, 22, 23, 24, 25]. Second, these methods rely on the use of catalysts generally based on expensive and rare metals. To address these issues, arylnitrile synthesis could largely benefit from the integration of new synthesis technologies [26, 27]. If alternative activation methods such as electrochemistry and photochemistry have already been explored (Figure 1c) [28, 29, 30, 31, 32, 33, 34], the developed reactions still involve the use of catalysts or electrolytes.

Finally, the most common nitrile sources, namely metal cyanides and silyl cyanides, usually raise toxicity issues and require specific handling precautions. Even if low‐toxicity nitrile sources such as acetonitrile have been studied, they still require the use of catalysts to be activated [35, 36, 37, 38, 39, 40]. Nowadays, the quest for a general and direct C‐H cyanation reaction of arenes that uses a low‐toxicity nitrile source and requires neither additive nor catalyst is still ongoing.

In this context, plasma should be considered as an attractive alternative activation for arylnitrile synthesis. Plasmas allow to generate reactive species at ambient temperature and atmospheric pressure in a gas phase without the use of a catalyst. While they are well known for gas phase synthesis [41, 42, 43, 44] and surface treatment [45, 46, 47, 48, 49], plasma–liquid processes have recently gained attention as new synthetic pathways in organic chemistry [50, 51, 52, 53, 54, 55, 56, 57, 58, 59, 60]. These processes rely on exposing a liquid phase to the reactive species generated by a plasma *via* mass exchanges at the gas–liquid interface [61, 62, 63].

Plasma‐assisted arene cyanation has already been studied in gas‐phase‐only systems using acetonitrile as the nitrile source (Figure 1d) [64, 65]. However, the complexity of the setup and its low efficiency led to the replacement of acetonitrile by cyanogen, which significantly improved arylnitrile yields but is highly toxic [66]. More recently, a gas–liquid system based on contact glow discharge electrolysis of a solution of arene in acetonitrile was explored [67]. This process relied on the dual role of the liquid phase as a reservoir of substrate and product, preventing the latter from overreacting and continuously providing reagents to the plasma. However, this strategy did not result in an increase in arylnitrile yields. Nevertheless, these early studies demonstrated the potential of plasma activation in arylnitrile synthesis.

In this work, we report an original approach to plasma‐assisted arene cyanation by using a gas–liquid microreactor. The mass transfer intensification enabled by the micrometric scale is a new promising strategy to improve arylnitrile yields when using acetonitrile as the nitrile source. Solutions of arenes in acetonitrile were exposed to an argon plasma in a biphasic gas–liquid segmented flow. The reactivity of radicals generated by acetonitrile plasma activation towards benzene was studied and used to propose a mechanism of benzene cyanation in this system. Exploring the effect of process parameters on this reaction produced similar results to previous gas–liquid plasma processes reported in the literature and shed light on the impact of gas phase mixing on the main products selectivity. Finally, the reaction scope was extended to different arenes and the formation of main nitriles was consistent with the proposed mechanism.

## 2 Results and Discussion

### 2.1 Experimental Setup

#### 2.1.1 Microreactor and Flow Setup

**FIGURE 2:**
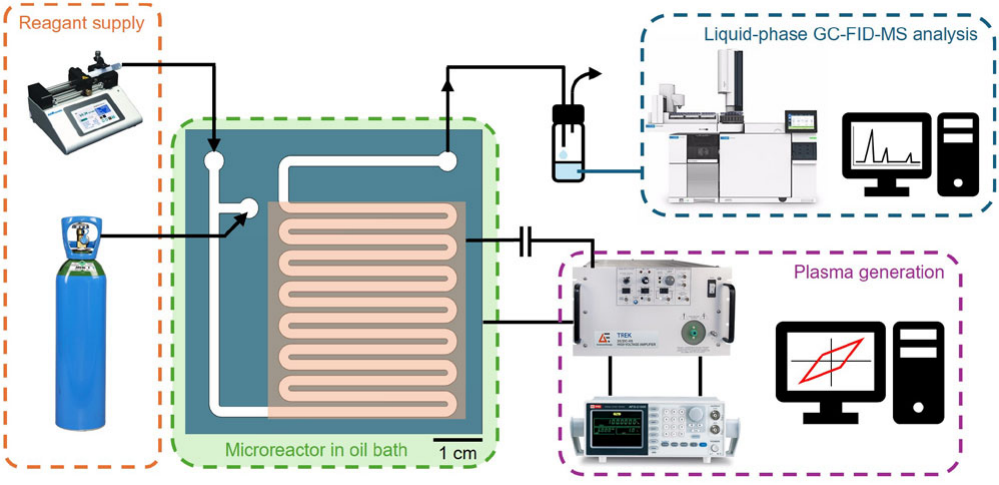
Setup used to perform arene cyanation by acetonitrile plasma activation.

The setup used to perform the experiments was extensively described in a previous work [68] and is schematized in Figure 2. The microreactor consists of a serpentine‐shaped channel of dimensions 55 cm × 1.84 mm × 210 μm (lenght × width × height) situated downstream of a T‐junction where a gas–liquid segmented flow is generated. The overall dimensions of the reactor are 6.1 cm × 6.1 cm × 410 μm. Its global volume is 1.5 mL for an internal volume of 0.3 mL. Gas and liquid were provided to the reactor through 1/32'' inner diameter PTFE tubings connected to the reactor using NanoPorts (Cluzeau Info Labo) fixed with epoxy resin (Loctite EA 9492).

The gas used was argon and was supplied to the microreactor from an Alphagaz 1 bottle (> 99.999 % purity) by a mass flow controller (Bronkhorst El‐Flow FG‐200CV, 0.70 mL/min capacity) with flow rates $Q_{g}$ from 0.05 mL/min to 0.25 mL/min. Liquid was supplied to the microreactor by a double syringe pump (KD Scientific Legato 180) with flow rates $Q_{l}$ from 0.05 to 0.25 mL/min. The liquid phase consisted of arene in acetonitrile solutions with concentrations from 1 10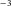 to 1 mol/L.

To avoid any cross‐contamination between successive experiments, the microreactor was washed for 5 min prior to each reaction with neat dimethylsulfoxide then neat acetone at 1 mL/min. It was then dried for 5 min under a 10 mL/min flow of argon. The biphasic flow was let to stabilize for 2.5 min before plasma ignition, then liquid collection was started 2.5 min after plasma ignition.

To ensure thermal regulation and electric insulation, the microreactor was immersed in an oil bath thermally controlled by a cryostat (Julabo Corio CD‐600F). The oil temperature varied from 278 to 328 K, and was set to 293 K unless stated otherwise.

#### 2.1.2 Plasma Generation and Monitoring

Plasma was generated by applying a sinusoidal high voltage of amplitude from 2.5 to 15 ${\mathrm{kV}}_{pp}$ and frequency from 0.25 to 2.5 kHz to copper electrodes deposited on each side of the microreactor. The high voltage supply comprised a low‐frequency generator (RS Pro AFG‐21025) connected to a voltage amplifier (Trek‐10/40A/HS). The ground electrode was connected to a capacitor to allow plasma power measurment via the Lissajous method (calculation details are available in the Supporting Information, see Section S1.1). Voltage and intensity were monitored using a Picoscope 3205D and the PicoScope 6 software.

#### 2.1.3 Chemical Analysis

The flow exiting the microreactor was collected in a vial where gas and liquid phases were separated by gravity. The liquid phase was analyzed afterwards by gas chromatography coupled to flame ionization detection (Agilent 7890B) and mass spectrometry (Agilent 5977B). The equipment was mounted with a HP‐5MS capillary column (Agilent J&W capillary GC columns, 30 m × 0.25 mm × 0.25 μm) and relied on a temperature‐programmed method (details are available in the Supporting Information, see Section S1.2). The injector temperature was set at 523 K and samples were injected in split mode with a split ratio of 100:1. Helium was used as the carrier gas at a constant flow rate of 1.0 mL/min. The MS detector operated in full scan mode with *m/z* in the range 30–500.

Products were identified using their retention time and by comparing their experimental mass spectrum with the NIST mass spectra database. All proposed structures had a matching score higher than 95 over 100 for all chromatograms. They were quantified by using ethyl acetate added as an external standard after the reaction and calibration curves (details are available in the Supporting Information, see Section S1.2). The different reaction caracteristics were calculated as follows. The substrate conversion was calculated as

(1) $$\text{Conversion}=\frac{n_{s,in}-n_{s,out}}{n_{s,in}}$$

with $n_{s,in}$ and $n_{s,out}$, the quantity of matter of substrate entering and exiting the reactor, respectively, measured by GC‐FID of the arene solution in acetonitrile and of the liquid phase collected at the microreactor outlet after reaction, respectively. Conversion represents the proportion of substrate that was consumed during the reaction. The product yield was calculated as

(2) $$\text{GC yield}=\frac{n_{p}}{\nu_{s}n_{s,in}}$$

with $\nu_{s}$ the number of substrate unit contained in the product, $n_{p}$ the quantity of matter of product in the liquid phase after reaction, and $n_{s,in}$ the quantity of matter of substrate entering the reactor, measured by GC‐FID of the liquid phase collected at the microreactor outlet and of the arene solution in acetonitrile respectively. Yield represents the overall reaction efficiency to produce a target product. In the whole manuscript, the term ”yield” refers to the GC yield. The liquid‐phase selectivity was calculated as

(3) $$\text{Liquid-phase selectivity}=\frac{n_{p,l}}{\Sigma n_{p,l}}$$

with $n_{p,l}$ the quantity of matter of a single product detected in the liquid phase derived from the substrate (products coming from solvent reactivity only are excluded). $\Sigma n_{p,l}$ thus represents the total quantity of matter of products present in the liquid phase derived from the substrate. The liquid‐phase selectivity represents the proportion of a single product compared to all products detected in the liquid phase.

### 2.2 Radicals Generated by Plasma

Segmented flows were generated in a microreactor using argon as the gas phase and arene in acetonitrile solutions as the liquid phase. The flow was composed of an alternance of gas bubbles and liquid slugs. Due to gas–liquid equilibrium at the interface and substrate dilution in the liquid phase, the gas phase should comprise approximately 95 mol% of argon, 5 mol% of acetonitrile vapor, and 0.1 mol% of substrate vapour (detailed calculations are available in the Supporting Information, see Section S2).

Plasma was generated inside bubbles by accelerating electrons with an external electric field. Collisions between the electrons and gaseous molecules lead to the formation of multiple reactive species, namely ions, radicals, photons, and excited species. Among these reactive species, radicals are considered to be at the basis of plasma‐assisted organic reactions [69]. However, in noble gas plasmas, it is recognized that radicals arise mainly from collisions between organic molecules and excited noble gas species, namely ${\mathrm{Ar}}^{*}$ in our system [69]. As acetonitrile predominates over the substrate in the gas phase, the vast majority of radicals should come from acetonitrile activation.

To create a radical from a collision between an acetonitrile molecule and an ${\mathrm{Ar}}^{*}$ atom, the energy of the excited specie has to be higher than the bond energy of the organic molecule to trigger bond dissociation. On one hand, the energy of the first excited species of argon is 11.5 eV [70]. On the other hand, the bond energies in acetonitrile are 4.0, 5.4, and 12.0 eV (or 390, 520, and 1160 kJ/mol) for C─H, C─C, and C≡N bonds, respectively [71, 72]. The C≡N bond therefore requires too much energy to be broken significantly, unlike the C─H and C─C bonds, and its dissociation can be considered negligible. Consequently, the main radicals formed by plasma activation of acetonitrile vapors are expected to be 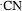 and 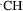 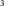 from C‐C bond dissociation, and 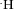 and 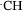 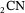 from C‐H bond dissociation.

### 2.3 Mechanism of Plasma‐Assisted Benzene Cyanation

**FIGURE 3:**
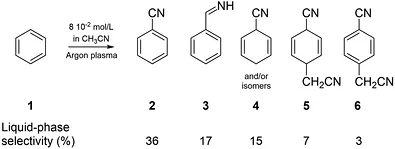
Main nitrogenous products formed after exposure of a benzene in acetonitrile solution to an argon plasma (10 ${\mathrm{kV}}_{pp}$, 1.5 kHz, 293 K, 8 10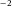 mol/L, $Q_{g}$ = $Q_{l}$ = 0.15 mL/min).

To understand how these radicals react with arenes, benzene was used as a model substrate. The reaction of a benzene in acetonitrile solution at contact with an argon plasma was performed. The analysis of the liquid phase by gas chromatography coupled with flame ionisation detection and mass spectrometry revealed the formation of multiple nitrogenous products summarized in Figure 3 (experimental details are available in the Supporting Information, see Section S3).

Henis et al. have already proposed a mechanism of arene cyanation that involves the addition of the 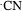 radical on the aromatic core, then rearomatization by elimination of a hydrogen atom [73]. The radical nature of this mechanism was confirmed by observing similar products from ICN photolysis in the presence of arenes, which produces only 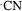 radicals as the reactive species [66]. However, the reactivity of the other radicals generated by acetonitrile plasma activation should also be considered.

Given the radical nature of the reaction, the formation of the main products is related to the reaction rate of radicals with the aromatic substrate. Once generated in the gas phase, radicals arising from acetonitrile plasma activation can undergo various reactions, namely, hydrogen atom transfer (HAT) or addition on an arene molecule, dimerization, and recombination with another radical. Calculations of the radical lifetimes using neat acetonitrile as the liquid phase revealed that the 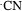 radical reacts the fastest and only in the gas phase, whereas the other radicals can diffuse to the liquid phase (detailed calculations are available in the Supporting Information, see Section S4). These results indicate that the main cyanation pathways involve a gas‐phase reaction of the arene with 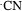 radicals.

The reaction rates of radicals generated by acetonitrile plasma activation on benzene are reported in Table 1. They show that 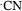 radicals are the fastest to react with benzene either by HAT or addition, yielding the phenyl 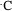 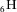 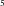 and the cyanocyclohexadienyle 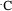 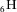 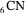 **7** radicals, respectively. Then, 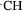 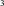, 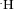, and 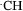 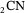 radicals have a similar reactivity and recombine with radicals coming from benzene.

**TABLE 1:** Reaction rate order of magnitude (in mol/(L·s)), at 283 K) of HAT, addition, dimerization and recombination reactions of the radicals generated by acetonitrile plasma activation with benzene.

Radical	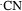	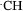 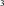	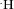	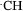 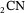
	**9**	**10**	**11**	**12**
HAT	10 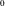	10 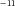	10 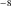	10 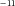
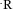 +SH = RH+ 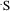				
Addition	10 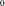	10 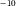	10 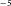	10 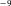
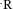 +S = ${\mathrm{RS}}^{.}$				
Dimerization	10 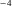	10 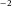	10 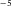	10 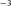
2 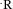 = $R_{2}$				
Recombination	10 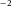	10 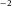	10 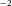	10 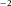
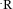 + 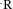 ' = RR'				

*Note*: The different reactions are also schematized (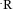, 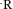
’: radicals, SH: hydrogen‐bearing substrate, S: addition‐site‐bearing substrate).

First, the HAT pathway was expected to yield benzonitrile **2**, benzylcyanide, and toluene. These products were not detected in the liquid phase despite significant amounts being reported in the literature [64, 65]. This discrepancy might be explained by the difference of benzene‐to‐acetonitrile ratio in this study, which is approximately two order of magnitudes lower. Phenyl radicals could therefore be quenched by reaction with acetonitrile faster than they recombine with radicals.

**FIGURE 4:**
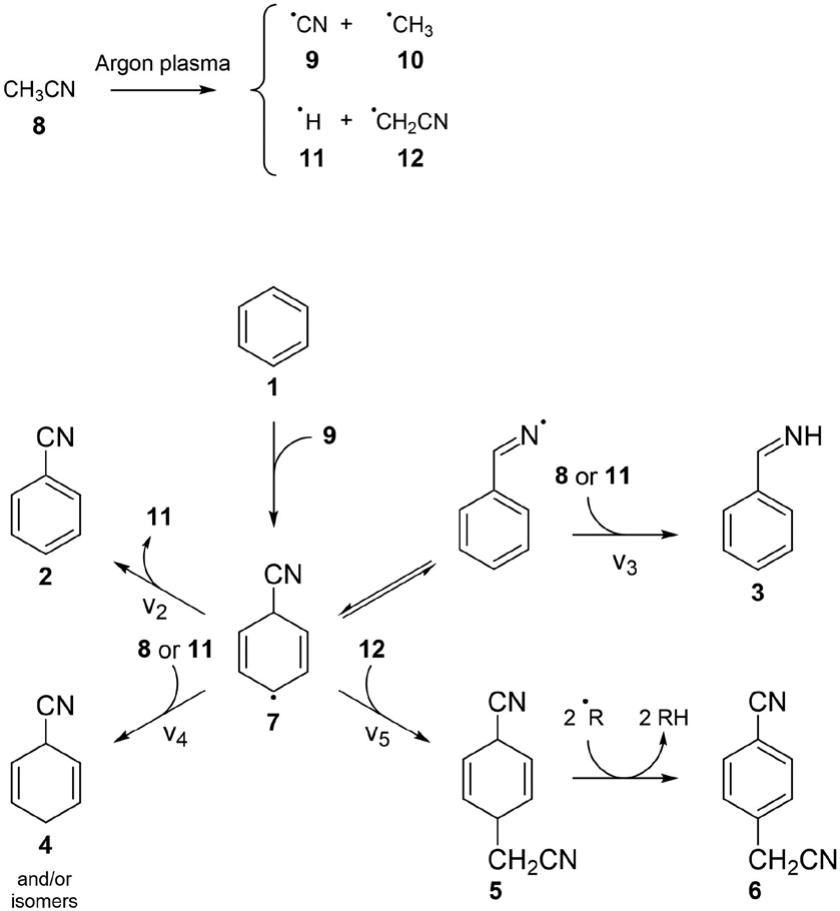
Proposed mechanism for the formation of the main nitrogenous compounds derived from benzene after the exposure of a benzene in acetonitrile solution to an argon plasma ($v_{x}$: formation rate of product **x**; 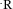: 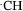 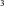, 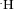 or 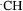 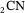 radical).

Second, the addition pathway was expected to yield **2** after rearomatization, cyclohexadienecarbonitrile **4**, methylcyclohexadienecarbonitrile, and cyanocyclohexadieneacetonitrile **5**. Based on this kinetic model, we propose the mechanism shown in Figure 4 to account for the formation of the main products of benzene cyanation by acetonitrile plasma activation. Among the products that could be formed via the addition pathway, only methylcyclohexadienecarbonitrile was not detected in the liquid phase. We hypothesized that 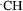 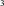 radical concentration was lower than that of other recombining radicals due to its higher dimerization rate, disfavoring the recombination reactions it was involved in.

However, two unexpected products were detected in the liquid phase after reaction and were identified as phenylmethanimine **3**, which formation and formal analysis at room temperature has already been reported [74, 75], and cyanophenylacetonitrile **6**. We hypothesized that **6** was formed by rearomatization of **5** in the liquid phase by HAT. This hypothesis was supported by the evolution of the selectivity of **5** and **6** with residence time (see Section 2.4 and detailed experimental data in the Supporting Information, see Section S5). However, this hypothesis could not be extended to the formation of **3** as its selectivity did not depend on residence time. In particular, compound **3** was not formed by overreaction of any product, ruling out its possible synthesis by addition of radical 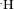 to benzonitrile. This observation was corroborated by the absence of products when benzonitrile was used as the substrate (Figure 9).

**FIGURE 5:**
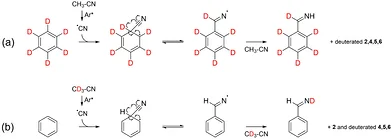
Benzene cyanation reactions by acetonitrile plasma activation performed using deuterated reagants (details of main deuterated products are available in the Supporting Information, see Section S6).

**FIGURE 6:**
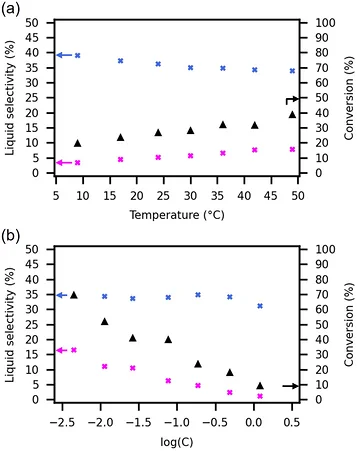
Evolution of benzene conversion (black triangles), benzonitrile selectivity (blue crosses) and benzonitrile yield (magenta crosses; arrows indicate the ordinate axis to use) as a function of temperature (a) or benzene concentration in the liquid phase (b, C in mol/L, log refers to the base 10 logarithmic function).

**FIGURE 7:**
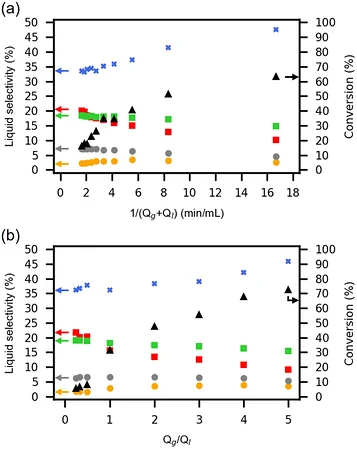
Evolution of benzene conversion (black triangles) and main products selectivity (blue crosses: **2**, red squares: **3**, green squares : **4**, gray circles: **5**, orange circles: **6**; arrows indicate the ordinate axis to use) as a function of 1/($Q_{g}$+$Q_{l}$) (a) or $Q_{g}$/$Q_{l}$ (b).

**FIGURE 8:**
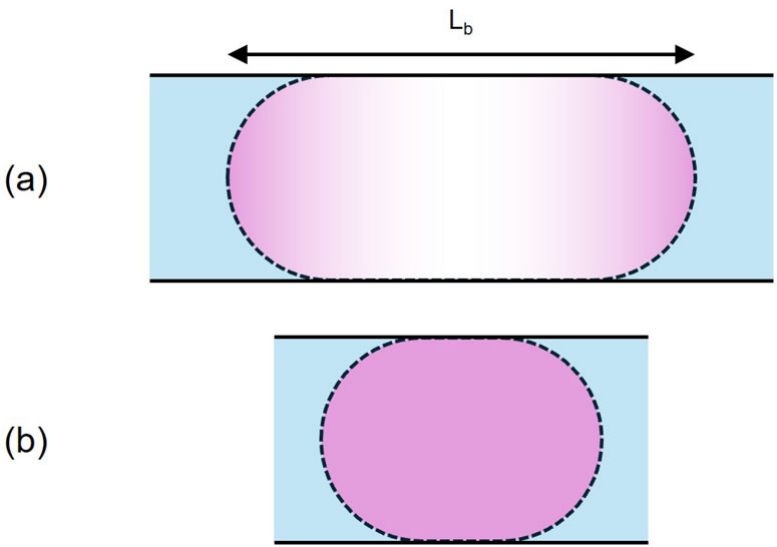
Schematic representation of a segmented flow (blue: liquid phase, white/pink: gas phase, dashed line: gas–liquid interface) generated with an arene in acetonitrile solution (pink: arene and acetonitrile vapors concentration scale in the gas phase) and argon in the hypothesis of a mass transfer limitation (a) or a perfect mixing (b) in the gas phase.

**FIGURE 9:**
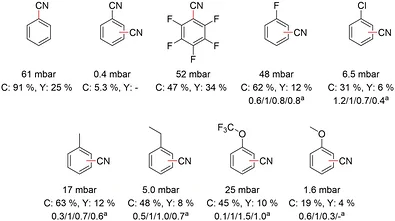
Substrate scope of arene cyanation by plasma activation of acetonitrile. For each experiment, the substrate vapor pressure at 283 K, conversion (C), and arylnitrile GC yield (Y) are indicated (10 ${\mathrm{kV}}_{pp}$, 1.5 kHz, 283 K, 8 10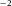 mol/L, $Q_{g}$ = 0.05 mL/min, $Q_{l}$ = 0.01 mL/min; 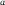: ratio of ipso/otho/meta/para isomer yields normalized to the ortho isomer, ‐: no arylnitrile detected in the liquid phase; created bonds are indicated in red; experiment details are available in the Supporting Information, see Section S9).

To understand the formation of **3**, deuterated reagents were used to incorporate deuterium atoms in the products, and the resulting modification of their mass spectra was analyzed (experimental details are available in the Supporting Information, see Section S6). The different reactions performed and mechanistic insights obtained are shown in Figure 5. Regarding phenylmethanimine, the use of deuterated benzene $C_{6}D$ 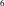 resulted in the integration of six deuterium atoms in **3** (Figure 5a). This suggested that a deuterium atom was transferred from the aromatic core to the C≡N bond after the addition of the 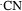 radical on $C_{6}D$ 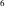. This transfer could occur between molecules of **2** and **7**. However, this possibility was ruled out as the addition of **2** to the liquid phase did not affect the selectivity of **3**, suggesting an intramolecular hydrogen transfer. When using deuterated acetonitrile ${\mathrm{CD}}_{3}\mathrm{CN}$, a single deuterium atom was incorporated in **3** on the nitrogen atom, evidencing the formation of an intermediate 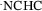 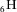 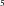 radical (Figure 5b). This radical then recombines with a 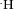 radical coming from acetonitrile plasma activation to yield **3**.

### 2.4 Optimization of Benzene Cyanation

Since cyanation of benzene by acetonitrile plasma activation could stand as an alternative direct route for benzonitrile synthesis, the effect of various process parameters on the reaction was investigated to optimize benzene conversion and benzonitrile selectivity. It was previously reported in the literature that relevant process parameters are plasma power, benzene concentration in the gas phase, residence time, and ratio between gas and liquid phases [54, 55, 56, 57, 58, 59, 60]. The corresponding parameters that can be modified are voltage and frequency, benzene concentration in the liquid phase and temperature, and gas and liquid flow rates $Q_{g}$ and $Q_{l}$, respectively. Each parameter was varied while maintaining the others constant, with the reference parameters being 10 ${\mathrm{kV}}_{pp}$, 1.5 kHz, 293 K, 8 10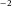 mol/L, $Q_{g}$/$Q_{l}$ = 1, and $Q_{g}$+$Q_{l}$ = 0.3 mL/min. As previously reported, the flow regime of the argon‐acetonitrile segmented flows generated remained unchanged in all experiments [68]. This was confirmed by observing the flow pattern under plasma using an iCCD camera (see the Supporting Information, Section S7).

The effect of plasma power and benzene concentration in the gas phase measured in our system was similar to what has been reported in the literature [57, 59, 60] (detailed experimental data are available in the Supporting Information, see Section S8). First, benzene conversion increased with plasma power without any modification of benzonitrile selectivity, resulting in a raise of benzonitrile yield (see Supporting Information, Figure S12). Second, increasing benzene concentration in the gas phase led to a small reduction of benzonitrile selectivity (Figure 6). This could be attributed to an increase in radical concentration in the gas phase that favors recombination reactions rather than the elimination pathway leading to benzonitrile formation. However, the effect of benzene concentration in the gas phase on benzene conversion depended on the ratio of benzene‐to‐acetonitrile concentrations in the gas phase α. Increasing temperature enabled to raise benzene concentration in the gas phase without modifying α because acetonitrile and benzene volatilities evolve similarly with temperature. This resulted in an increase in benzene conversion and ultimately in benzonitrile yield (Figure 6a). Conversely, increasing benzene concentration in the liquid phase led to an increase in α and therefore to a decline in benzene conversion, and ultimately in benzonitrile yield (Figure 6b).

Finally, the effect of residence time and gas‐to‐liquid ratio was investigated. Figure 7 shows the evolution of benzene conversion and main products selectivity as a function of 1/($Q_{g}$+$Q_{l}$) and $Q_{g}$/$Q_{l}$. As expected and previously reported in the literature, increasing either the residence time or the gas‐to‐liquid ratio improved benzene conversion [54, 55, 56, 57, 58, 59, 60].

Surprisingly, increasing the residence time induced a rise in benzonitrile selectivity from 33% to 48% at 1/($Q_{g}$+$Q_{l}$) = 1.67 min/mL and 1/($Q_{g}$+$Q_{l}$) = 16.7 min/mL, respectively (Figure 7a). However, no modification of the selectivity towards benzonitrile was observed when the contact time was increased by recirculating the reaction medium (see the Supporting Information, Section S5). This could be explained by the low vapor pressure of the products **2**–**6** compared to benzene, which limits their further reaction, in accordance with previous results [55, 56, 57, 60].

Modifying the gas‐to‐liquid ratio resulted in a similar evolution of selectivities, with an increase of benzonitrile selectivity from 36% to 46% at $Q_{g}$/$Q_{l}$ = 0.2 and $Q_{g}$/$Q_{l}$ = 5, respectively (Figure 7b). We hypothesized this evolution could be explained by a variation in the characteristic mass transfer times within the flow. This change in selectivity results from a competition between the first‐order reaction producing **2** from **7** and the second‐order reactions forming products **3**–**6** (Figure 4). Since these reactions occur in the gas phase and second‐order reaction rates depend on the concentration of the involved species, a difference in gas phase mixing could alter species distribution in the gas phase and thus affect selectivities.

Segmented flows are known to drastically improve mixing within gas and liquid phases because the alternation of bubbles and slugs creates recirculation vortices inside both phases, allowing a mixing by convection together with diffusion [76, 77]. The characteristic convection and diffusion times in the gas phase can be defined as $t_{c}$ ∼ $L_{b}$/U and $t_{d}$ ∼ $L_{b}^{2}$/D, respectively, with $L_{b}$ the bubble length, U the bubble speed, and *D* the diffusion coefficient in the gas phase. Since U ∼ ($Q_{g}$+$Q_{l}$) and $L_{b}$ ∼ $Q_{g}$/$Q_{l}$ [68, 78], results obtained by varying gas and liquid flow rates suggest that benzonitrile selectivity was improved with increasing characteristic mass transfer times, and so with decreasing the efficiency of gas phase mixing.

A slow mass transfer induces a concentration gradient of the various vaporized species in the gas phase (Figure 8a), which promotes the first‐order reaction ($v_{2}$, Figure 4) and therefore the formation of **2**. Conversely, intensifying mass transfer by shortening bubbles or increasing flow rates homogenizes species concentration in bubbles (Figure 8b), which promotes second‐order reactions ($v_{3}$ to $v_{5}$, Figure 4). Using segmented flows therefore allowed to tune the enrichment of the gas phase with organic compound vapors, which resulted in an increase of the target product selectivity. This study is the first to rely on segmented flows to perform a gas–liquid plasma process, and so is the first to report the effect of mixing on selectivity in such a process.

Through screening the process parameters, the experimental conditions to reach the highest benzonitrile selectivity were identified as 10 ${\mathrm{kV}}_{pp}$, 1.5 kHz, 283 K, 8 10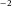 mol/L, $Q_{g}$ = 0.05 mL/min, and $Q_{l}$ = 0.01 mL/min. A benzene conversion of 91% and a benzonitrile liquid selectivity of 62 % for a yield of 25 % were achieved, which is promising given the short residence time of approximately 10 s used in this study. This optimisation was conducted on a 0.1 mg scale for fast and reproducible results.

To demonstrate the relevance of this new preparation method of arylnitriles, the space‐time yield of benzonitrile synthesis was also investigated. The space‐time yield based on the reactor volume reached 65 g/(L·h), which was validated on a 10 mg scale under the following experimental conditions: 15 ${\mathrm{kV}}_{pp}$, 2.5 kHz, 283 K, 1.2 mol/L, $Q_{g}$ = 0.3 mL/min, and $Q_{l}$ = 0.3 mL/min. Given the low reactor internal volume and the low collection time used, these results support the potential of this new process for arylnitrile preparation. Therefore, a larger scale synthesis of arylnitriles using our method seems possible by increasing either the reactor internal volume or the collection time, or by parallelizing multiple microreactors.

### 2.5 Substrate Screening for Plasma‐Assisted Cyanation of Arenes

Based on these encouraging results, the scope of plasma‐assisted cyanation was broadened to other arenes. The main nitriles formed for all arenes used were the corresponding arylnitriles and benzonitrile, as shown in Figure 9. These products are expected to form via a mechanism similar to the one of benzene cyanation [73], since the reactivity of radicals generated by acetonitrile plasma activation is expected to be similar toward arenes and benzene. Arylnitriles should therefore be formed by 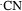 addition on the aromatic core, then rearomatization by hydrogen elimination.

It was observed that conversion increased with substrate vapor pressure for substituents of similar nature, supporting the hypothesis that the reaction occurred in the gas phase. In particular, the absence of dicyanobenzenes in benzene cyanation could be explained by benzonitrile low vapor pressure. In addition, arylnitrile regioisomers were formed during the reaction. The ratio of the latter were similar to works reported in the literature and did not exhibit any significant electronic effects, which is consistent with the proposed radical nature of the mechanism [65, 67].

Finally, no reaction of radicals on the substituents was observed: for example, no product arising from HAT with the ethyl substituent of ethylbenzene was detected in the liquid phase after reaction. This could indicate that 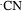 radical addition on C‐${\mathrm{sp}}^{2}$ is faster than HAT with C‐${\mathrm{sp}}^{3}$, but kinetic data lack to corroborate this hypothesis.

## 3 Conclusion

This study presents a plasma flow reactor for the C‐H cyanation of arenes conducted at ambient temperature and pressure without catalyst. The reaction proceeds efficiently via plasma activation of acetonitrile. Under optimized conditions, a benzene conversion of 91% and a benzonitrile liquid selectivity of 62% were achieved, resulting in a 25% yield. The methodology was applied to various arenes and conversion was found to strongly depend on substrate vapor pressure, supporting the hypothesis that the reaction occurs in the gas phase. Kinetic studies combined with experiments using deuterated reagents provided mechanistic insights explaining the formation of the different products observed. Although plasma activation in flow offers advantages, it faces challenges such as limited selectivity due to the high reactivity of involved species and limited productivity. These issues can be addressed by designing novel reactor geometries that increase the gas–liquid interfacial area and developing advanced plasma sources with better control over reactive species generation.

## Conflicts of Interest

The authors have no conflict to declare.

## Supporting information


**Supporting File**: chem71101‐sup‐0001‐SuppMat.pdf.
