# Supplementary material for: Catalyst‐Free Cyanation of Arenes by Acetonitrile in a Plasma–Liquid Microreactor
Source: Chemistry. 2026 May 13;32(32):e71101. doi: 10.1002/chem.71101 (PMC13505769; doi:10.1002/chem.71101)
Supplement: Supplementary file 1 [file CHEM-32-e71101-s001.pdf]

Chemistry – A European Journal

Supporting Information

Catalyst-Free Cyanation of Arenes by Acetonitrile in a Plasma-Liquid Microreactor

Pierre Dedieu, Gabriel Morand*, Stéphanie Ognier, Alain Favre-Réguillon, Michael Tatoulian

Contents

1 Materials and methods **3**
1.1 Plasma generation and monitoring . . . 3
1.2 Chemical analysis . . . 3

2 Calculation of approximate gas phase composition **5**

3 Experimental details on benzene cyanation by acetonitrile plasma activation **6**
3.1 Neat acetonitrile exposed to an argon plasma . . . 6
3.2 Benzene in acetonitrile exposed to an argon plasma . . . 8

4 Calculation of radical reaction rate **10**

5 Experimental details on the recirculation of the liquid phase after benzene cyanation by acetonitrile plasma activation **12**

6 Experimental details on reactions to elucidate the formation of phenylmethanimine and cyanophenylacetonitrile **13**
6.1 Benzene-d6 in acetonitrile exposed to an argon plasma . . . 13
6.2 Benzene in acetonitrile-d3 exposed to an argon plasma . . . 14

7 Images of argon-acetonitrile segmented flows in absence and in presence of plasma **15**

8 Experimental details on benzene concentration in the liquid phase, temperature, and plasma power optimisation **16**

9 Experimental details on arene screening **17**

9.1 Cyanation of benzonitrile 17
9.2 Cyanation of hexafluorobenzene 18
9.3 Cyanation of fluorobenzene 19
9.4 Cyanation of chlorobenzene 20
9.5 Cyanation of toluene 21
9.6 Cyanation of ethylbenzene 22
9.7 Cyanation of trifluoromethoxybenzene 23
9.8 Cyanation of anisole 24

1 Materials and methods

1.1 Plasma generation and monitoring

The electrical setup used to generate plasma within the microreactor is described in figure SI.1a. Plasma was generated by applying a sinusoidal high voltage to the copper electrodes deposited on each side of the microreactor. The ground electrode was connected to a 96.9 nF capacitor to allow plasma power measurement *via* the Lissajous method. Voltage and intensity were monitored using a PicoScope 3205D and the PicoScope 6 software.

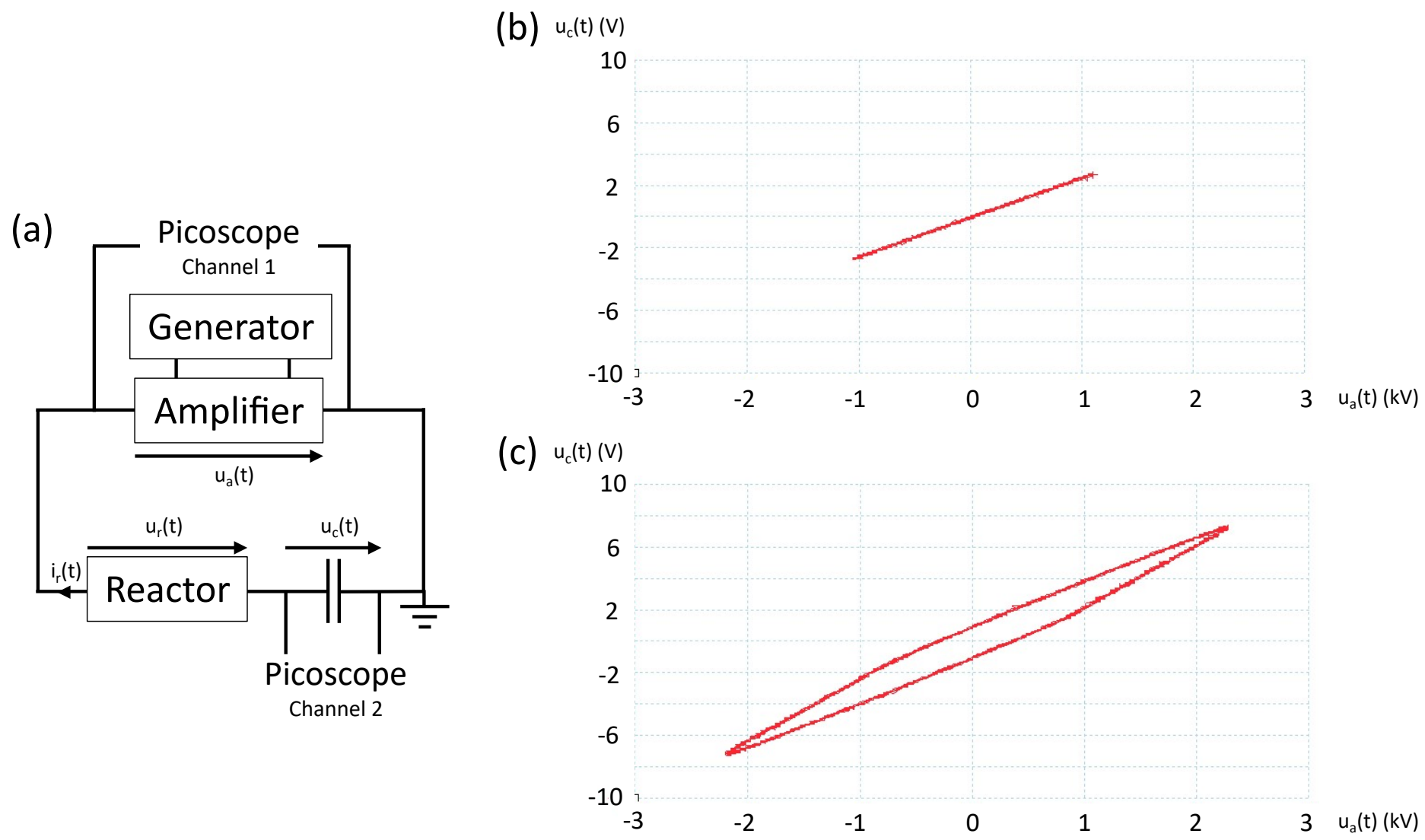

Fig. SI. 1 Electrical setup used for plasma experiments (a) and Lissajous plot before (b) and after (c) plasma ignition in the reactor.

The Lissajous method relies on the use of a capacitor placed in series with the reactor in order to monitor the electrical current flowing through it. The instantaneous power in the reactor $p(t)$ can be expressed as a function of the voltage applied to it $u_r(t)$ and the current that flows through it $i_r(t)$:

$$p(t) = u_r(t) \cdot i_r(t) \tag{SI.1}$$

The reactor capacity was around 150 pF and could be assumed negligible compared to the capacity $C = 96.9$ nF of the capacitor used. Thus the electrical current could be calculated by measuring the voltage on the capacitor $u_c(t)$:

$$i_r(t) = C\frac{du_c(t)}{dt} \tag{SI.2}$$

The voltage on the capacitor varied from 1 V to 30 V and was considered negligible compared to the voltage applied to the reactor, which was therefore approximated to the voltage delivered by the amplifier $u_a(t)$. The plasma power P was measured by averaging the temporary power over a period of the sinusoidal voltage T:[1]

$$P = \frac{1}{T}\int_0^T u_r(t)i_r(t)dt = \frac{1}{T}\int_0^T u_a(t)du_c(t) \tag{SI.3}$$

Plasma power was monitored continuously and averaged over the collection time of an experiment.

The plot of $u_c(t)$ as a function of $u_a(t)$ is called a Lissajous plot and allows to visualize when the gas breakdown occurs. Figures SI.1(b) and SI.1(c) show an example of a Lissajous plot before and after plasma ignition respectively. The breakdown voltage was measured by slowly increasing the voltage until the Lissajous plot shape changed. In our system, the breakdown voltage of argon at contact with acetonitrile was measured at 3.2 kV ± 0.5 kV_{pp} independently of fluidic conditions.

1.2 Chemical analysis

The flow exiting the microreactor was collected in a vial where gas and liquid phases were separated by gravity. The liquid phase was analyzed afterwards by gas chromatography coupled to flame ionisation detection (Agilent 7890B) and mass spectrometry (Agilent 5977B). GC-FIS-MS analysis was conducted using a temperature-programmed method. The oven temperature was initially set to 50 °C and held for 1 min. It was then increased at a rate of 40 °C/min to 170 °C, where it was maintained for 0.5 min. A second ramp raised the temperature at a rate of 20 °C/min to 250 °C, followed by a final ramp at a rate of 40 °C/min up to 300 °C, which was held for 2 min. The total run time of the method was 11.75 min.

Products were identified using their mass spectrum and retention time. They were quantified by using ethyl acetate added as an external standard after the reaction and calibration curves:

$$n_p = \alpha \cdot n_{es} \frac{A_p}{A_{es}} \tag{SI.4}$$

with n_p and n_{es} the quantity of matter of product and external standard respectively, A_p and A_{es} the area of the product and external standard FID signals respectively, and α the response factor of the product towards the external standard. The latter was determined using calibration curves realised prior to the experiments or was estimated to the one of a molecule with a similar structure. Table SI.1 gathers the different reponse factors used and their method of determination. Benzene calibration curve is available in figure SI.2, other calibration curves are available in section 9. The signals corresponding to different regioisomers are discriminated using their boiling point and their response factors are considered equal. To estimate arylnitrile response factors, it was observed that benzonitrile has a response factor 0.77 times that of benzene. The same correspondance factor was applied to the arene response factor to obtain the one of the deriving arylnitriles.

Molecule	Response factor	Method of determination
Benzene	0.444	Calibration curve
Benzonitrile	0.342	Calibration curve
Phenylmethanimine	0.34	Response factor of benzonitrile
Cyclohexadienecarbonitrile	0.34	Response factor of benzonitrile
Cyanocyclohexadieneacetonitrile	0.32	Response factor of terephthalonitrile
Cyanophenylacetonitrile	0.32	Response factor of terephthalonitrile
Hexafluorobenzene	0.380	Calibration curve
Pentafluorobenzonitrile	0.3	Estimated from hexafluorobenzene
Fluorobenzene	0.422	Calibration curve
Fluorobenzonitrile	0.3	Estimated from fluorobenzene
Chlorobenzene	0.387	Calibration curve
Chlorobenzonitrile	0.3	Estimated from chlorobenzene
Toluene	0.343	Calibration curve
Methylbenzonitrile	0.3	Estimated from toluene
Ethylbenzene	0.3	Single point calibration
Ethylbenzonitrile	0.2	Estimated from ethylbenzene
Trifluoromethoxybenzene	0.5	Single point calibration
Trifluoromethoxybenzonitrile	0.4	Estimated from trifluoromethoxybenzene
Anisole	0.373	Calibration curve
Methoxybenzonitrile	0.3	Estimated from anisole

Table SI. 1 Response factors towards ethyl acetate used to calculate species quantity of matter from the GC-FID analysis of the liquid phase.

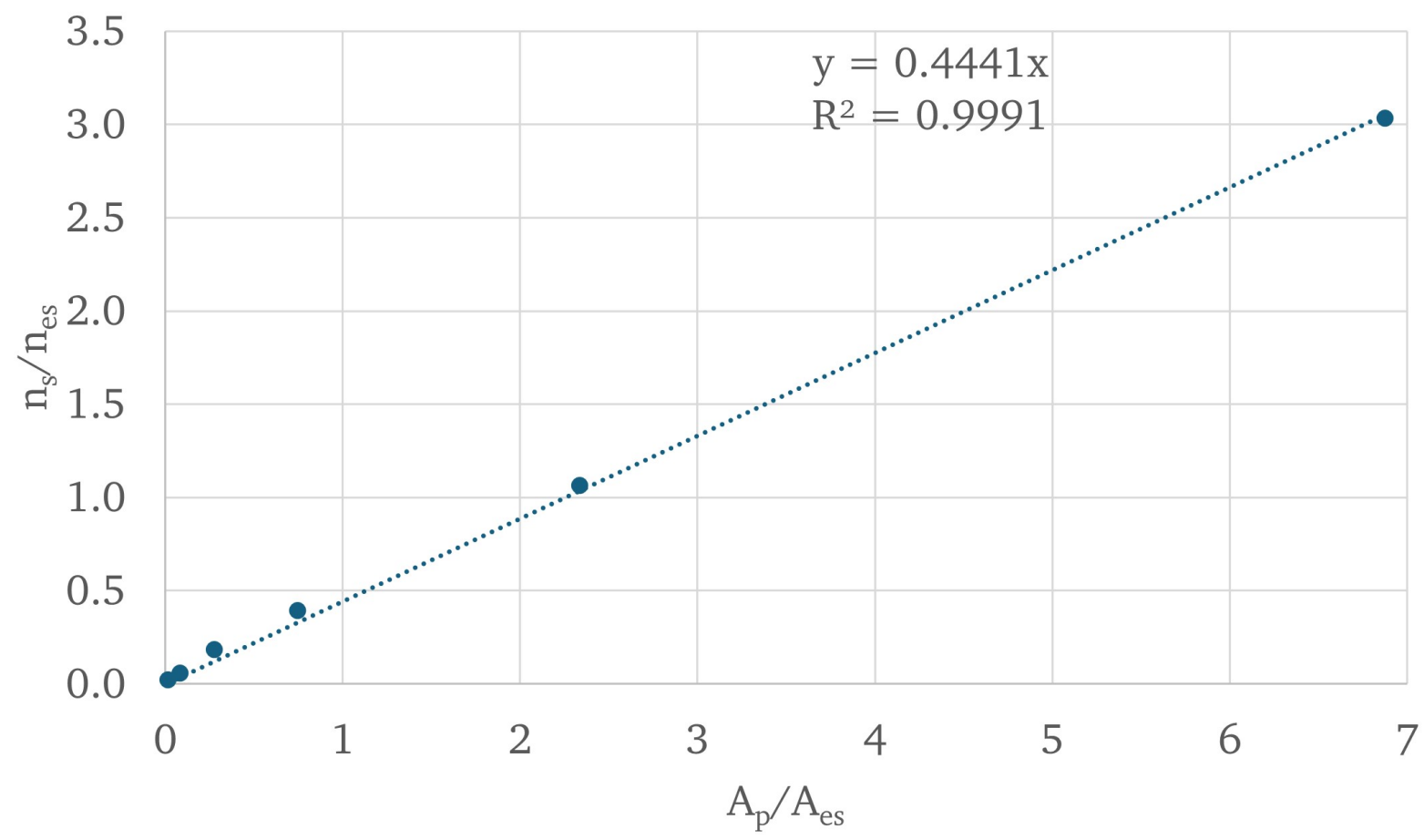

Fig. SI. 2 Calibration curve of benzene using ethyl acetate as the external standard.

2 Calculation of approximate gas phase composition

In this work, segmented flows were generated using argon as the gas phase and an arene in acetonitrile solution as the liquid phase. Due to liquid-vapour equilibrium at the gas-liquid interface, the gas phase is not composed of pure argon but also of arene and acetonitrile vapours. In a previous work, we showed the pressure drop across the microreactor in argon-acetonitrile segmented flows remained lower than 25 mbar in the fluidic conditions range explored.[2] The pressure inside bubbles in this work was therefore considered equal to 1 atm. An approximate gas phase composition can then be computed by considering the gas phase is saturated of the different organic compounds. Using Raoult law, the partial pressure P_{org} of acetonitrile or the arene in the gas phase can be expressed as:

$$P_{org} = x^{l}_{org} \cdot P^{sat}_{org} \tag{SI.5}$$

with x^{l}_{org} the molar fraction of the considered organic compound in the liquid phase and P^{sat}_{org} its vapour pressure. In the case of benzene and using the data available in the literature[3] at a temperature of 10 °C and at a benzene concentration of 10^{-1} mol/L, equation SI.5 yields an acetonitrile partial pressure of 57 mbar and a benzene partial pressure of 0.3 mbar. In terms of order of magnitude, the gas phase is therefore composed of 95 mol% argon, 5 mol% acetonitrile vapours and 0.1 mol% benzene vapours.

3 Experimental details on benzene cyanation by acetonitrile plasma activation

3.1 Neat acetonitrile exposed to an argon plasma

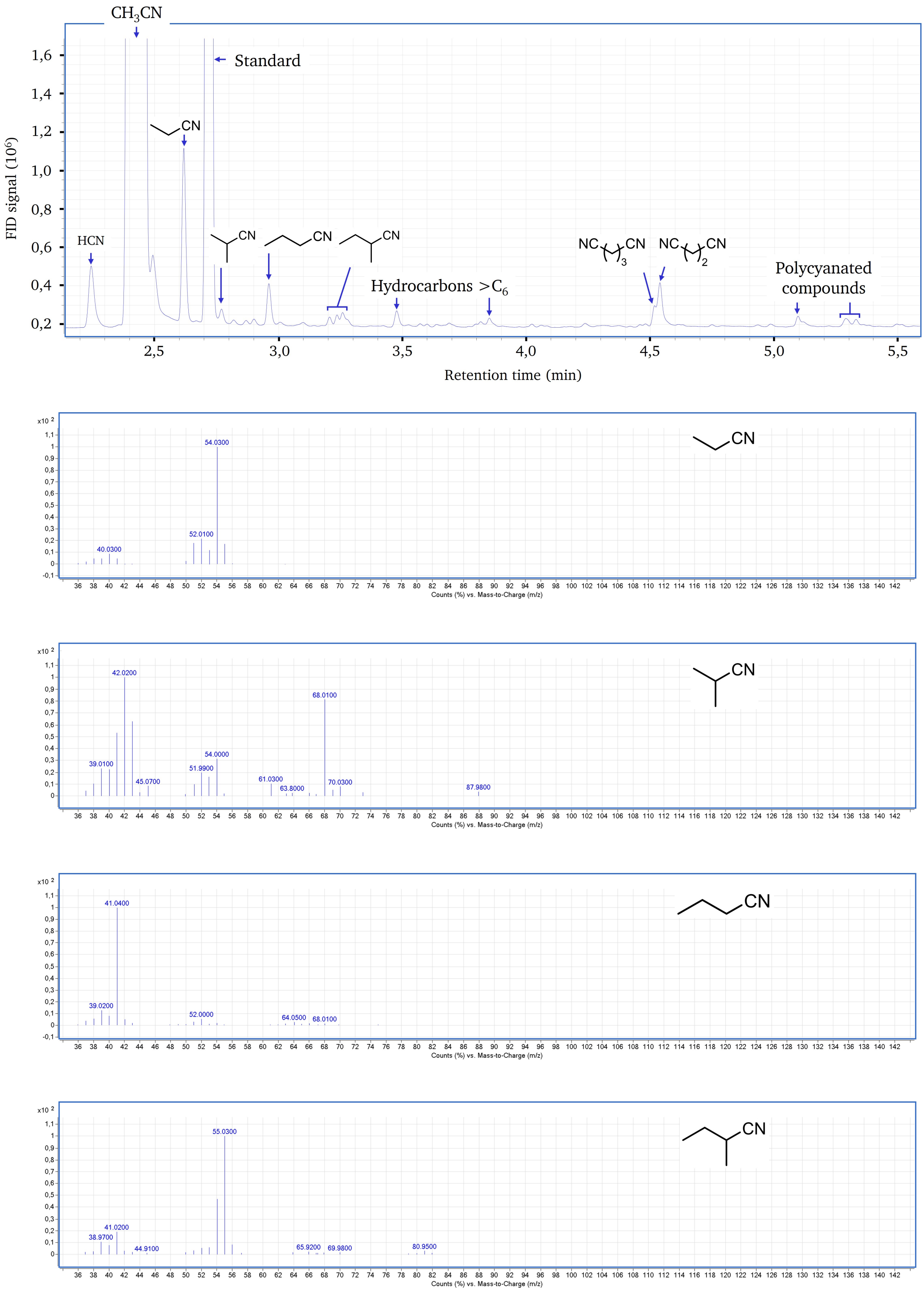

Fig. SI. 3 GC-FID chromatogram of the liquid phase collected at the microreactor outlet after exposure of neat acetonitrile to an argon plasma (10 kV_{pp}, 1.5 kHz, 10 °C , Q_g = 0.05 mL/min, Q_l = 0.01 mL/min). The mass spectra of the main product are also provided.

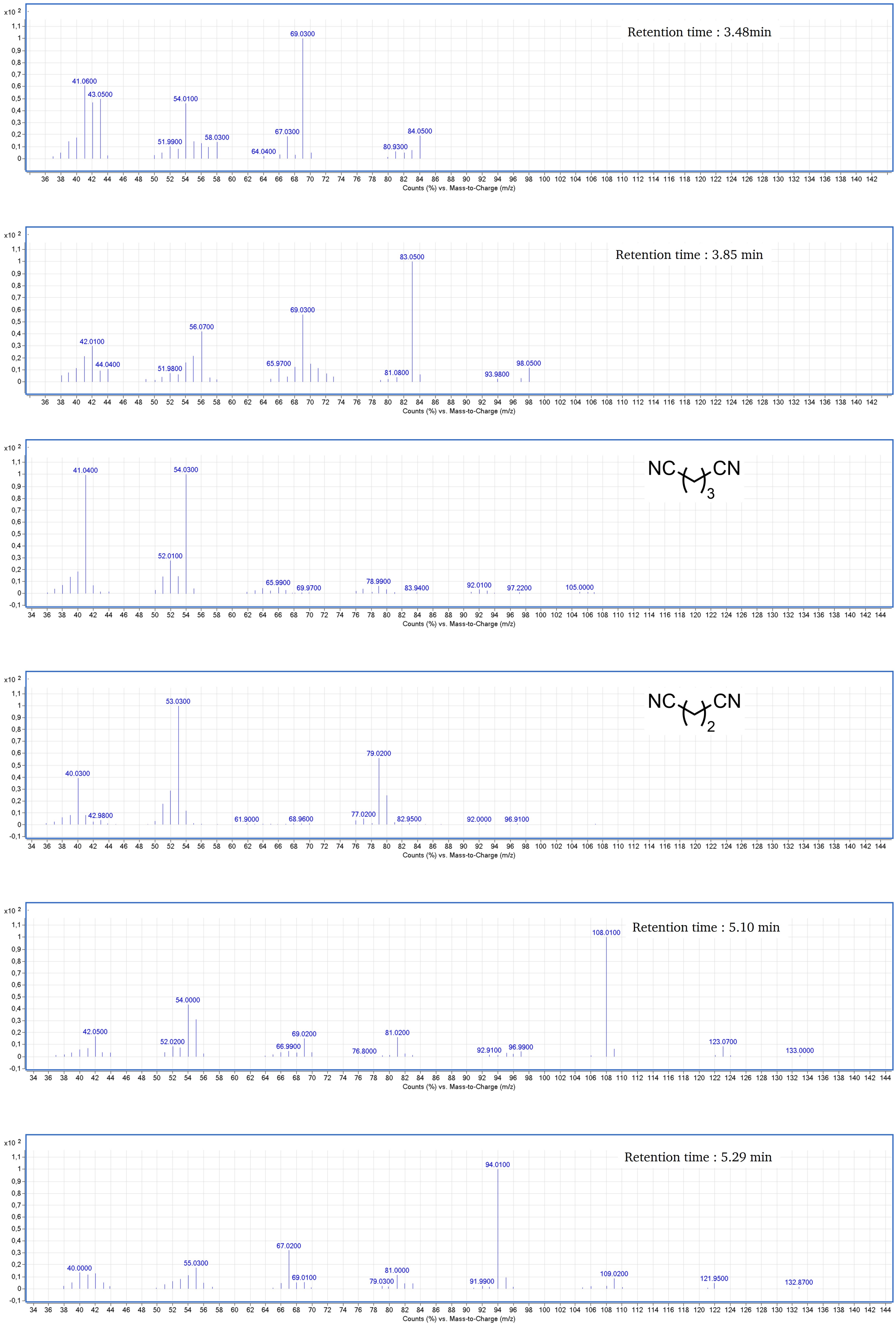

Fig. SI. 4 Continuation of Figure SI.4.

3.2 Benzene in acetonitrile exposed to an argon plasma

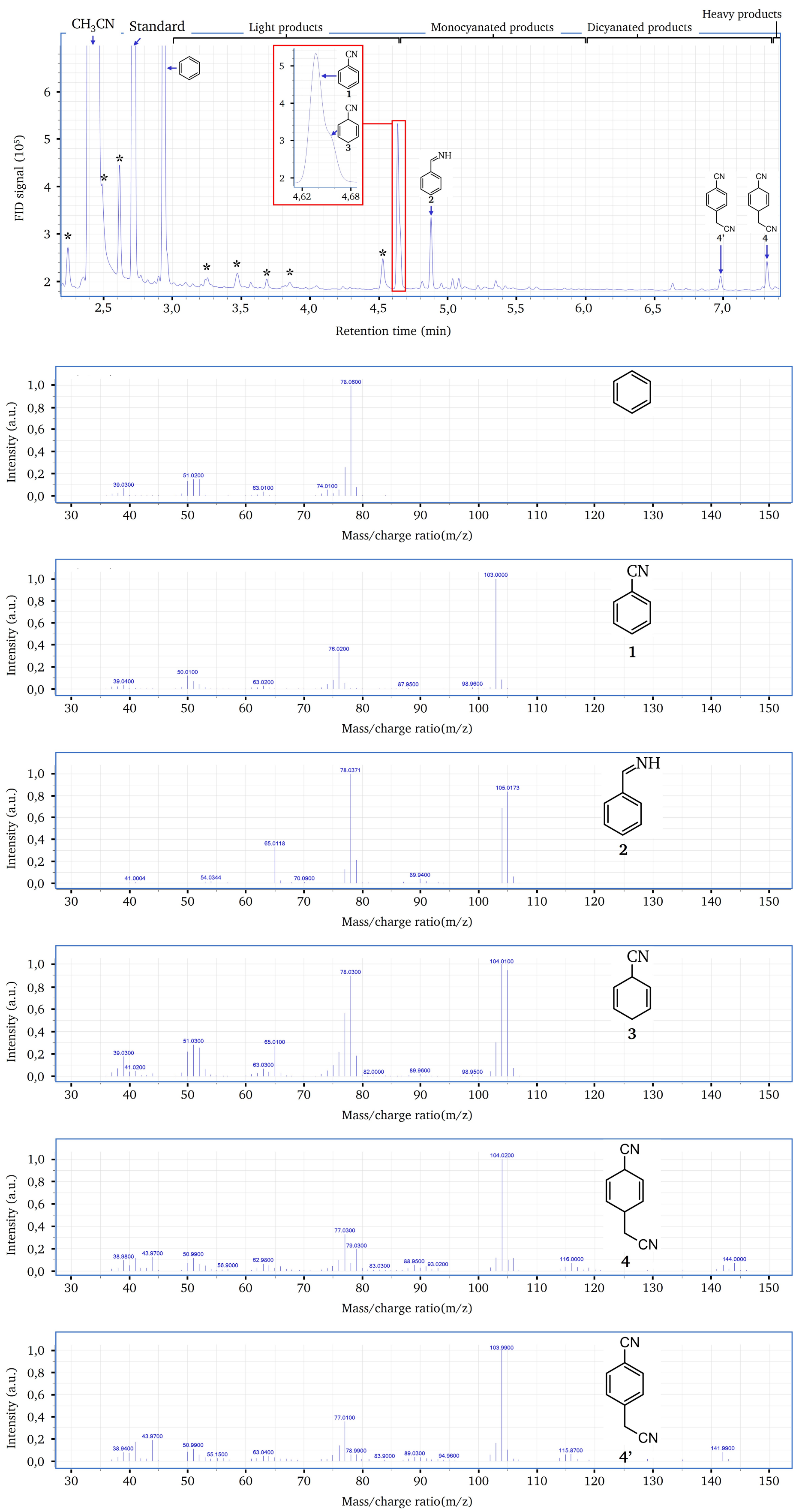

Fig. SI. 5 GC-FID chromatogram of the liquid phase collected at the microreactor outlet after exposure of a benzene in acetonitrile solution to an argon plasma (10 kV_{pp}, 1.5 kHz, 10 °C , 8 10^{-2} mol/L, $Q_g = Q_l = 0.15$ mL/min). Products deriving from the reactivity of neat acetonitrile are indicated with a star. The mass spectra of the main products are also provided.

In the case of benzene cyanation by acetonitrile plasma activation, the mass spectra of each product was investigated. Based on the structural information provided by the MS analysis, products could be categorized in four distinct groups:

- light products : these products are more volatile than benzonitrile. Their retention time spread from 2.93 min to 4.65 min. Their structure usually contains an hydrogenated aromatic core or methyl groups. Their response factor was assimilated to that of benzene.
- Cyanated compounds : these products have a similar volatility to benzonitrile. Their retention time spread from 4.65 min to 6.00 min. Their mass spectra shows the loss of a m/z = 27 fragment caracteristic of the nitrile group. Their response factor was assimilated to that of benzonitrile.
- Dicyanated compounds : these products have a similar volatility to terephtalonitrile. Their retention time spread from 6.00 min to 7.35 min. Their mass spectra shows the loss of two m/z = 27 fragments caracteristic of the nitrile group. Their response factor was assimilated to that of terephtalonitrile.
- Heavy compounds : these products less volatile than terephtalonitrile. Their retention time was higher than 7.35 min. Their response factor was assimilated to that of terephtalonitrile.

Benzene cyanation by plasma activation of acetonitrile was performed ten times to estimate the uncertainties on conversion and main products selectivity. The results are summarized in figure SI.6, where the uncertainty is equal to the standard deviation.

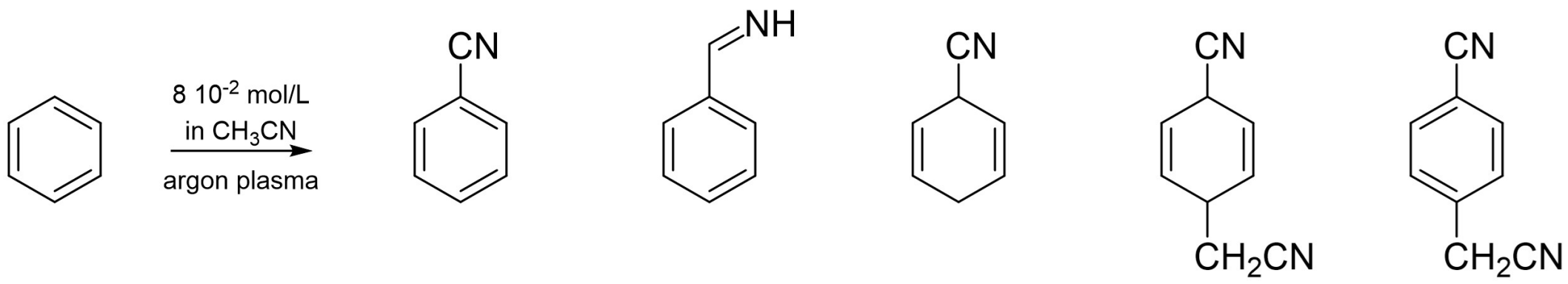

	1	2	3	4	4'
Liquid-phase selectivity (%)	35.7 ± 1.2	17.3 ± 1.2	15.4 ± 1.0	7.0 ± 0.5	3.3 ± 0.6

Conversion: 33.7 ± 3.9 %

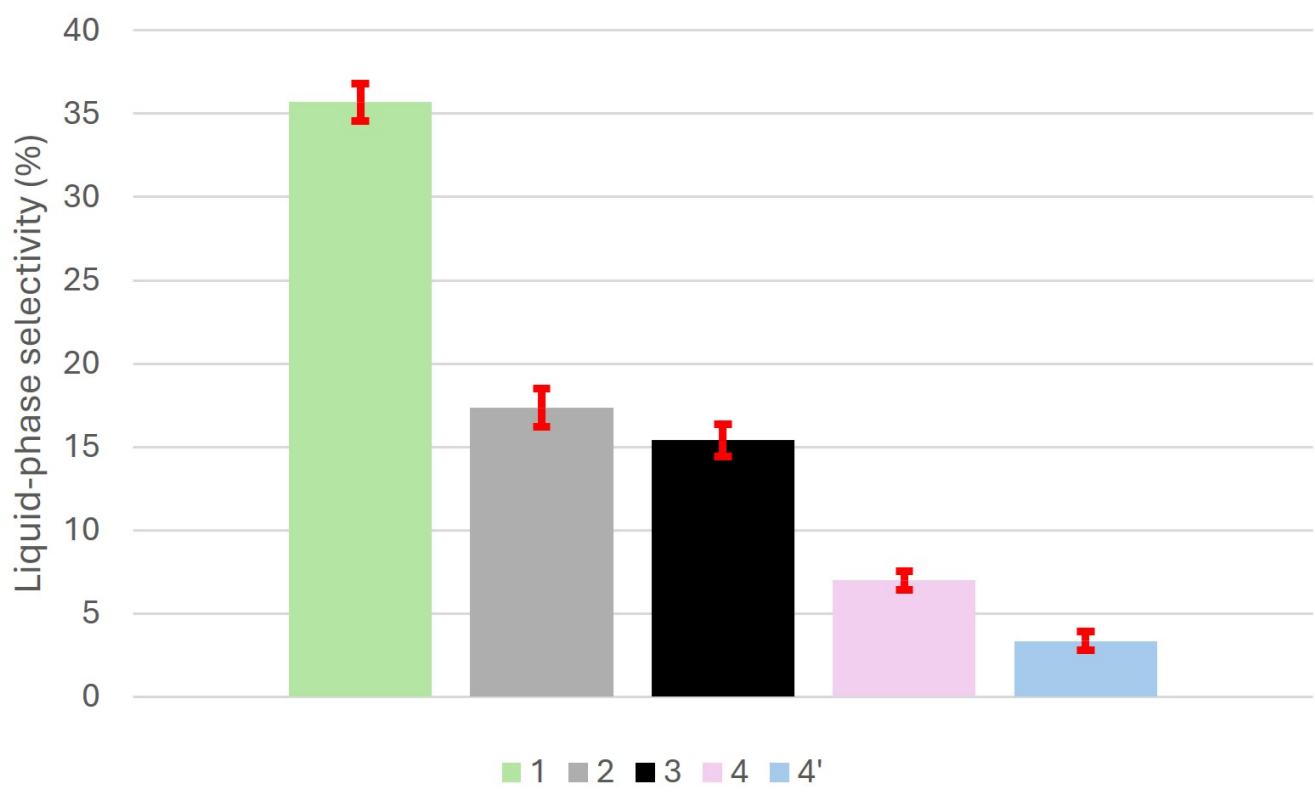

Fig. SI. 6 Average values and uncertainties on conversion and main products liquid-phase selectivity for benzene cyanation by acetonitrile plasma activation (10 kV_{pp}, 1.5 kHz, 10 °C , 8 10^{-2} mol/L, $Q_g = Q_l = 0.15$ mL/min).

4 Calculation of radical reaction rate

To understand how radicals generated by acetonitrile plasma activation react, their reaction rate on the different organic compounds present in the gas phase must be calculated. These reactions can be categorized in two distinct groups : first, reactions implying only radicals, namely recombination between two distinct radicals and dimerisation of a radical; second, reactions implying a radical and an organic molecule, namely abstraction, addition and substitution. For molecules studied in this work, kinetic constants reported in the literature show that abstraction reactions mostly involve the transfer of a hydrogen atom. Also, substitution reactions are much slower than the other reactions and so were neglected.[3] The reactions considered were therefore H-abstraction, addition, dimerisation, and recombination, and the considered radicals were $^{\bullet}CN$, $^{\bullet}CH_3$, $^{\bullet}H$ and $^{\bullet}CH_2CN$.

These reactions are elementary acts either of second or third-order, which allows to express the reaction rates v_r as follows:

$$v_r = k[R][S] \text{ for second-order reactions and} \tag{SI.6}$$

$$v_r = k[R][S][M] \text{ for third-order reactions,} \tag{SI.7}$$

with k the kinetic constant of the reaction, [R] and [S] the radical and substrate concentrations respectively, and [M] the total concentration of gaseous species (*i.e.* 43 mol/m^3 at atmospheric pressure and 10 °C). Based on kinetic data available in the literature, dimerisation reactions are third-order whereas the other reactions are second-order.[3]

However, kinetic data of reactions involving acetonitrile and the considered radicals are scarce in the literature. Acetonitrile was therefore assimilated to propene as the chemical structure of both molecules is quite similar and kinetic data for propene are much more abundant. Kinetic constants available in the literature for both molecules on a set of different radicals were of the same order of magnitude, except for the addition reaction which is approximately five order of magnitudes slower on acetonitrile than propene.[3] The reaction rate of the considered radicals with acetonitrile was therefore approximated to that with propene except for the addition reaction, which was multiplied by 10^{-5}. In the case of a kinectic data lack, the kinetic constant of a reaction implying molecules with a structure as close as possible to the considered species was chosen.

Finally, the composition of the gas phase needs to be estimated. Calculations detailed in section 2 allows to estimate the concentration of the different species involved to 2.4 mol/m^3 of acetonitrile vapours and 1.3 10^{-2} mol/m^3 of benzene vapours. Radicals will therefore mainly be consumed by reaction with acetonitrile vapours and these reactions will determinate their respective half-time. Last, previous works estimated the production of radicals during a plasma discharge at 100 ppm of the activated compound.[4,5] In this work, only acetonitrile was considered to be activated by plasma due to its prevalence in the gas phase. The concentration of $^{\bullet}CN$, $^{\bullet}CH_3$, $^{\bullet}H$ and $^{\bullet}CH_2CN$ radicals was therefore considered equal to 2.4 10^{-4} mol/m^3.

The reaction rates of $^{\bullet}CN$, $^{\bullet}CH_3$, $^{\bullet}H$ and $^{\bullet}CH_2CN$ radicals with acetonitrile are reported in table SI.2.

Radical	Abstraction	Addition	Dimerisation	Recombination
$^{\bullet}H$	10^{0}	10^{-2}	10^{-2}	10^{1}
$^{\bullet}CH_3$	10^{-5}	10^{-9}	10^{1} [a]	10^{1}
$^{\bullet}CN$	10^{5} [b]	10^{0} [b]	10^{-2}	10^{1}
$^{\bullet}CH_2CN$	10^{-7} [c]	10^{-13} [d]	10^{0} [a]	10^{1}

Table SI. 2 Reaction rate order of magnitude (in mol/(m^3·s), at 10 °C) of abstraction, addition, dimerisation and recombination reactions of the radicals generated by acetonitrile plasma activation with acetonitrile ([a] : second-order, [b] : substrate is ethylene, [c] : reaction of radical $^{\bullet}CH_2CCH$ on ethanol, [d] : reaction of radical $^{\bullet}CH_2CCH$ on acetylene). Kinetic data are extracted from the NIST database.[3]

Based on these calculations, $^{\bullet}CN$ radicals react mainly by H-abstraction whereas $^{\bullet}CH_3$, $^{\bullet}H$ and CH_2CN radicals react mainly by recombination with other radicals. As these reactions are second-order, the radical half-time t_l can be estimated as:

$$t_l = \frac{1}{2k_{fast}[R]} \tag{SI.8}$$

with k_{fast} the kinetic constant of the fastest reaction for each radical and [R] its concentration. This calculation also allows to estimate the distance on which radicals can diffuse in the gas phase before they are consumed by chemical reactions d_{diff} as:

$$d_{diff} = (t_l \cdot D)^{\frac{1}{2}} \tag{SI.9}$$

with D the diffusion coefficient of the radical in the gas phase, considered equal to 10^{-5} m^2/s. Equations SI.8 and SI.9 allow to estimate $^{\bullet}CN$ radical half-time to 1 ns and its diffusion length to 0.1 μm. As the dimensions of the channel are at least three order of magnitudes higher, $^{\bullet}CN$ radicals are considered to react only in the gas phase. Regarding $^{\bullet}CH_3$, $^{\bullet}H$ and $^{\bullet}CH_2CN$ radicals, their half-time is estimated to 10 μs and their diffusion length to 10 μm, which is only one order of magnitude smaller than the smallest channel dimension. These radicals were therefore considered able to diffuse to the liquid phase.

A same reasoning can be used to determine the radicals main reactivity towards benzene instead of acetonitrile. The kinetic constants used therefore implied benzene instead of propene. Recombination reactions then involve radicals generated by acetonitrile plasma activation and radicals coming from benzene. However, the most concentrated radicals coming from benzene are produced by the reaction of radicals generated by acetonitrile plasma activation with benzene itself due to the prevalence of acetonitrile in

the gas phase. Their concentration was approximated to the one of these radicals, *i.e.* $2.4\ 10^{-4}$ mol/m^3. The reaction rates of $^{\cdot}$CN, $^{\cdot}CH_3$, $^{\cdot}$H and $^{\cdot}CH_2CN$ radicals with benzene are reported in table SI.3.

Radical	Abstraction	Addition	Recombination
$^{\cdot}$H	10^{-5}	10^{-2}	10^{1}
$^{\cdot}CH_3$	10^{-8}	10^{-7}	
$^{\cdot}$CN	10^{3}	10^{3}	
$^{\cdot}CH_2CN$	10^{-8} [a]	10^{-6} [b]	

Table SI. 3 Reaction rate order of magnitude (in mol/(m^3·s), at 10 °C) of abstraction, addition, dimerisation and recombination reactions of the radicals generated by acetonitrile plasma activation with benzene ([a] : addition of $^{\cdot}CH_3$ radical, [b] : addition of $^{\cdot}CH_2CCH$ radical). Kinetic data are extracted from the NIST database.[3]

5 Experimental details on the recirculation of the liquid phase after benzene cyanation by acetonitrile plasma activation

To ensure no product is formed by the overreaction of another product, the liquid phase collected after a reaction was recirculated three times. This experiment consisted in using the collected liquid phase as intrant of the next experiment. The GC-FID chromatograms had the classical shape previously shown in section 3.2. Figure SI.7 shows the conversion and the main products selectivity as a function of the recirculation number.

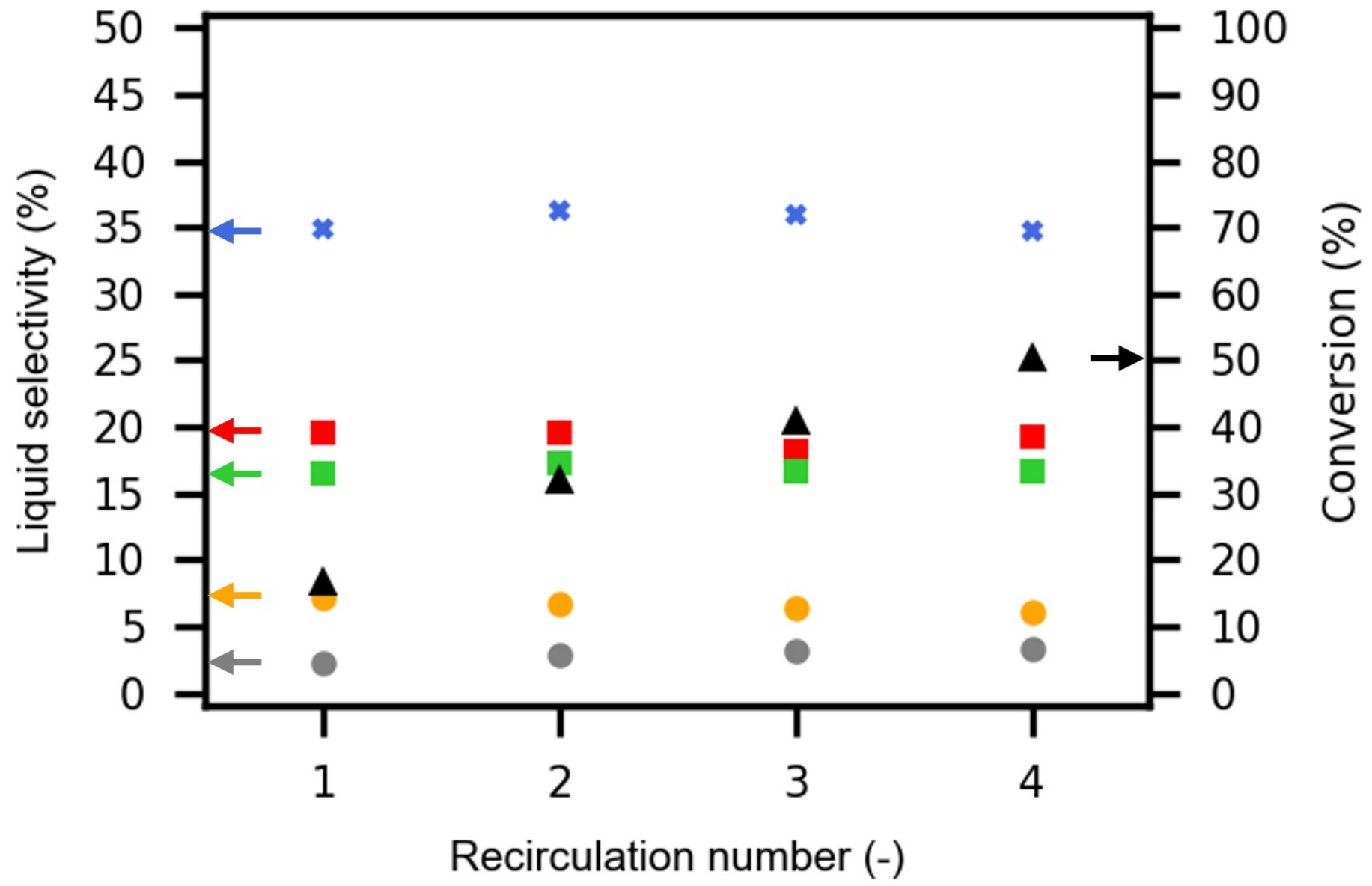

Fig. SI. 7 Evolution of benzene conversion (black triangles) and main products selectivity (blue crosses : 2, red squares : 3, green squares : 4, grey circles : 5, orange circles : 6; arrows indicate the ordinate axis to use) as a function of recirculation number (10 kV_{pp}, 1.5 kHz, 10 °C , 8 10^{-2} mol/L, $Q_g = Q_l = 0.30$ mL/min).

6 Experimental details on reactions to elucidate the formation of phenylmethanimine and cyanophenylacetonitrile

6.1 Benzene-d6 in acetonitrile exposed to an argon plasma

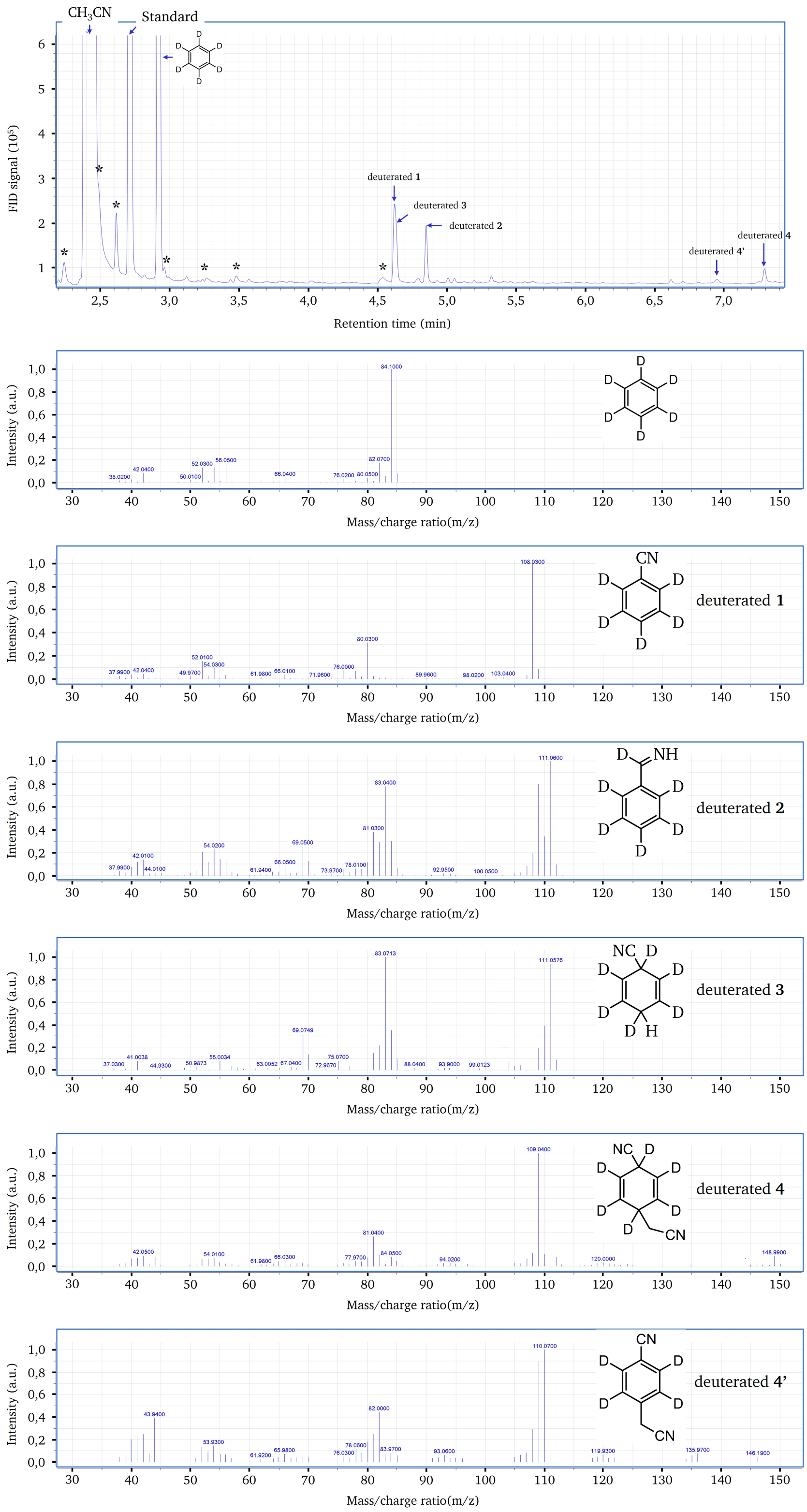

Fig. SI. 8 GC-FID chromatogram of the liquid phase collected at the microreactor outlet after exposure of a benzene-d6 in acetonitrile solution to an argon plasma (10 kV_{pp}, 1.5 kHz, 10 °C , 8 10^{-2} mol/L, $Q_g = Q_l = 0.15$ mL/min). Products deriving from the reactivity of neat acetonitrile are indicated with a star. The mass spectra of the main products are also provided.

6.2 Benzene in acetonitrile-d3 exposed to an argon plasma

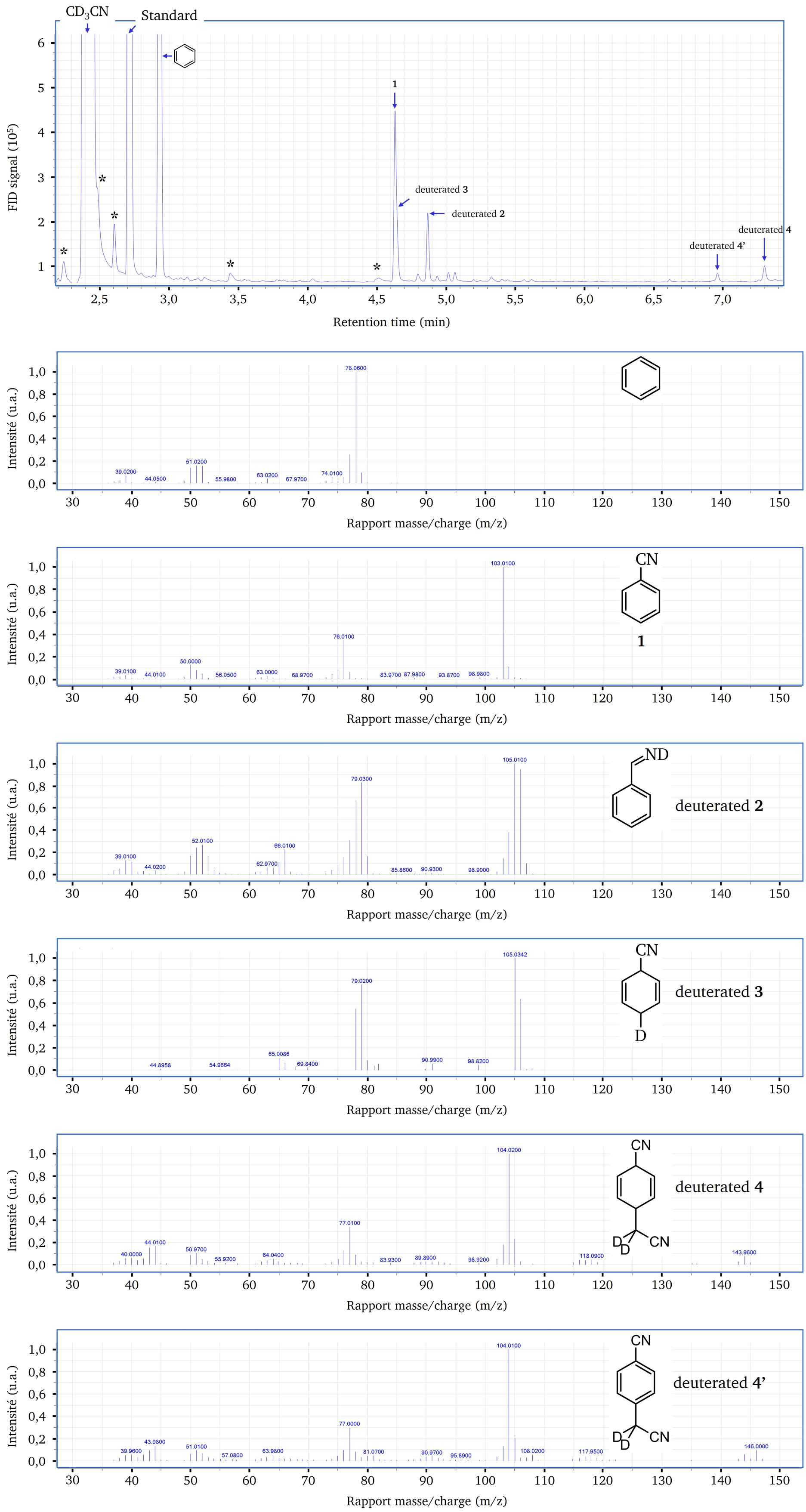

Fig. SI. 9 GC-FID chromatogram of the liquid phase collected at the microreactor outlet after exposure of a benzene in acetonitrile-d3 solution to an argon plasma (10 kV_{pp}, 1.5 kHz, 10 °C , 8 10^{-2} mol/L, $Q_g = Q_l = 0.15$ mL/min). Products deriving from the reactivity of neat acetonitrile-d3 are indicated with a star. The mass spectra of the main products are also provided.

7 Images of argon-acetonitrile segmented flows in absence and in presence of plasma

To visualise the gas-liquid flow during experiments, a lamp (Leica LED3000 BLI) was placed beneath the reactor and was coupled to an imaging camera (Pixelink PL-B781U) placed above the reactor. The transmitted light was observed by using transparent ITO electrodes deposited on each side of the reactor (the deposition process is described below).

To visualise the light emitted by plasma, the experimental setup was placed in a dark environment. An iCCD camera (Princeton Instruments Kuro back illuminated sCMOS) was placed above the reactor and coupled to the LightField software. Pictures were taken with a pose time of 20 ms after an automatic color balance of the white and black scale.

ITO electrodes were deposited on the reactor using the hybrid magnetron sputtering (HSPT520, Plasmionique Inc.) from Institut Pierre Gilles de Gennes clean room. Prior to ITO deposition, the reactor surface was cleaned with an oxygen/argon plasma at 50 W for 5 min (5 mTorr, $Q_{Ar} = 2\ Q_{O_2} = 20$ mL min^{-1}, substrate holder rotating at 5 rpm). The atmosphere was replaced by pure argon flowing at 30 mL min^{-1} at a pressure of 9 mTorr. The deposition process started with a pre-sputtering step where ITO target power was slowly increased from 15 W to 50 W at 5 W min^{-1} while maintaining the target shutter closed. This step aimed at eliminating target pollution and avoiding thermal stress on the target. Once at 50 W, the target shutter was opened for a deposition time of 10 min (substrate holder rotating at 5 rpm, substrate to target distance of 30 mm). After ITO target temperature decrease and chamber pressurising, the process was repeated to deposit an electrode on the other side of the reactor. After deposition of both electrodes, the reactor was submitted to a thermal treatment at 400 °C for 2 h. After thermal annealing, the thickness of the electrodes was 50 nm and the transmittance of the electrodes was 90 % at 493 nm, while the resistivity of the electrodes was measured to 1.0 ± 0.1 kΩ/cm.

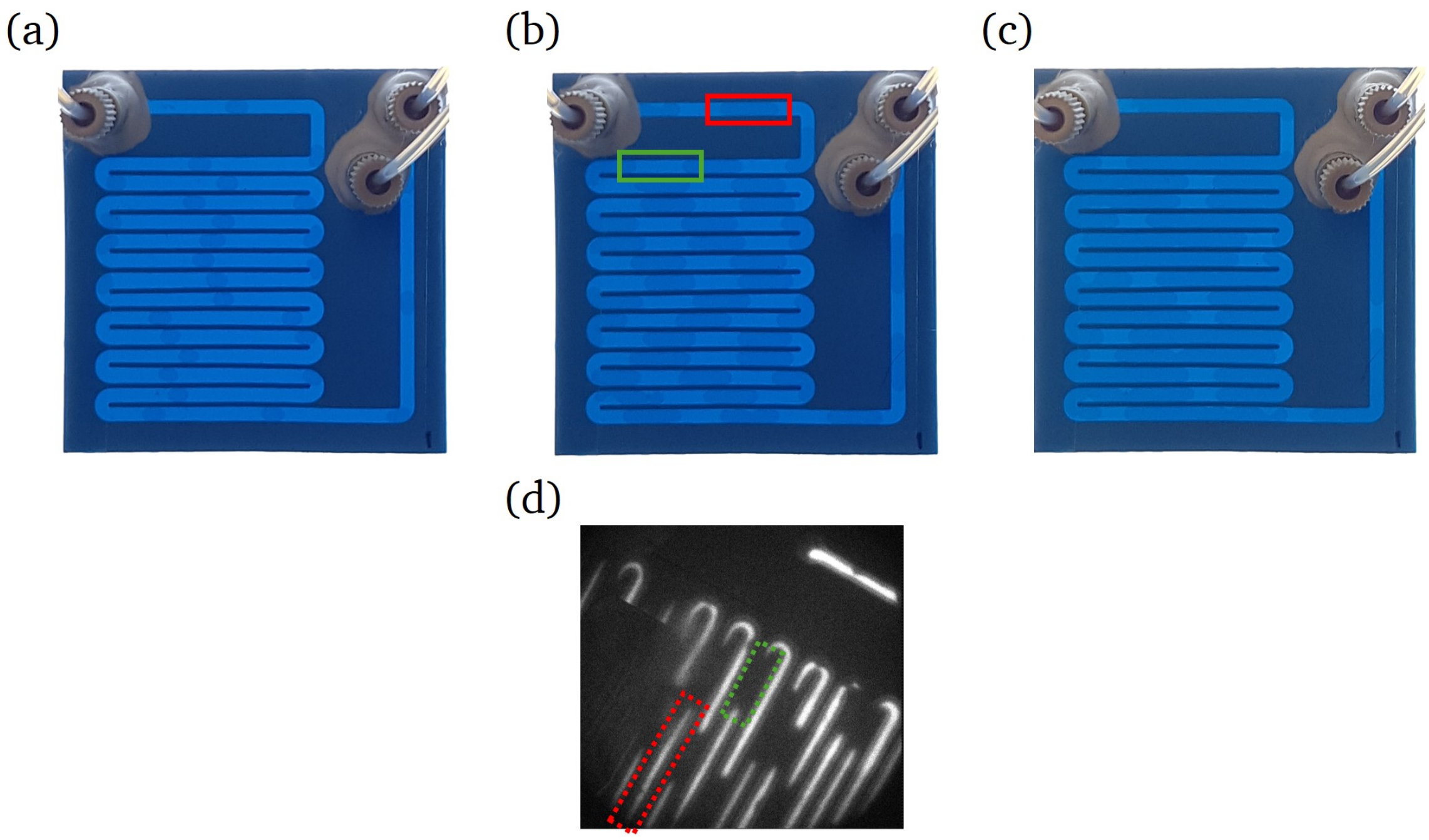

Fig. SI. 10 Pictures of the segmented flows generated in the microreactor using argon as the gas phase and an arene solution in acetonitrile as the liquid phase ($Q_g+Q_l = 0.3$ mL/min; a: $Q_g/Q_l = 0.2$; b: $Q_g/Q_l = 1$; c: $Q_g/Q_l = 5$; d: $Q_g/Q_l = 1$ in presence of plasma, 10 kV$_{pp}$, 1.5 kHz, light intensity in black and white scale). For readability reasons, a gas bubble and a liquid slug are highlighted in red and green respectively (continuous line: absence of plasma, dotted line: presence of plama).

8 Experimental details on benzene concentration in the liquid phase, temperature, and plasma power optimisation

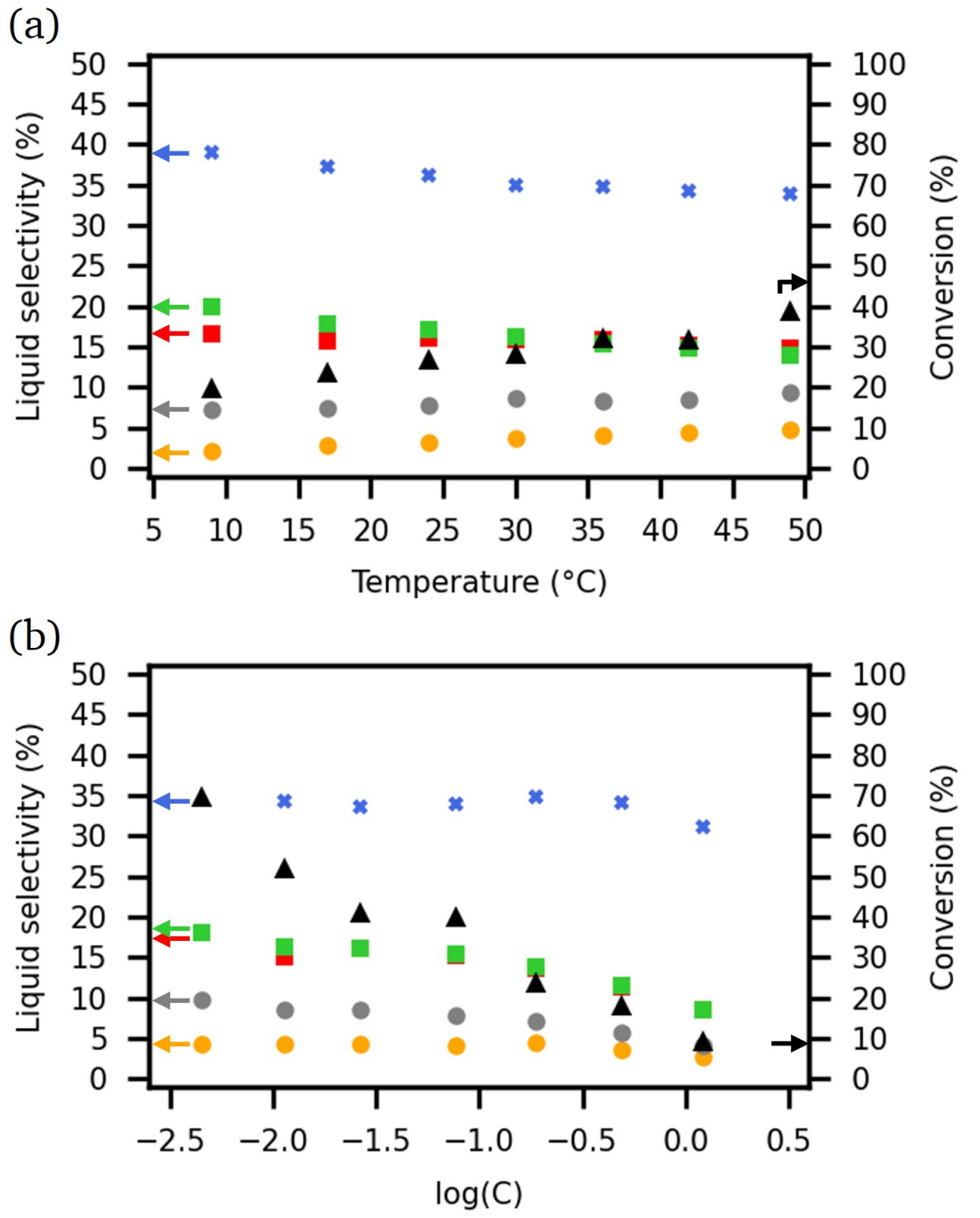

Fig. SI. 11 Evolution of benzene conversion (black triangles) and main products selectivity (blue crosses : 2, red squares : 3, green squares : 4, grey circles : 5, orange circles : 6; arrows indicate the ordinate axis to use) as a function of benzene concentration in the liquid phase (a, at 20 °C, C in mol/L, log refers to the base 10 logarithmic function) or temperature (b, at 8 10^{-2} mol/L) (10 kV_{pp}, 1.5 kHz, $Q_g = Q_l = 0.15$ mL/min).

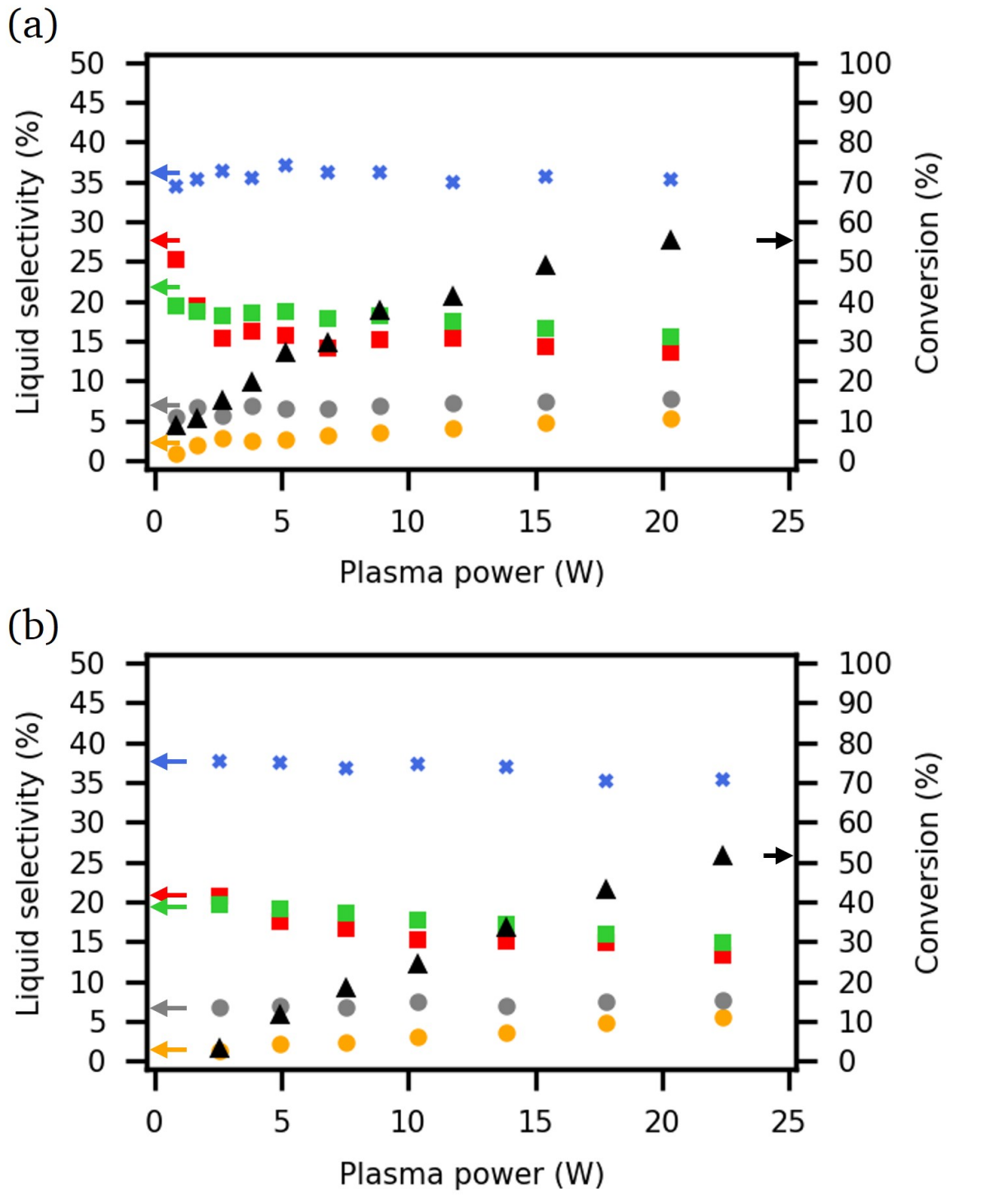

Fig. SI. 12 Evolution of benzene conversion (black triangles) and main products selectivity (blue crosses : 2, red squares : 3, green squares : 4, grey circles : 5, orange circles : 6; arrows indicate the ordinate axis to use) as a function of plasma power when frequency (a, at 10 kV_{pp}) or voltage (b, at 1.5 kHz) was varied (20 °C, 8 10^{-2} mol/L, $Q_g = Q_l = 0.15$ mL/min).

9 Experimental details on arene screening

9.1 Cyanation of benzonitrile

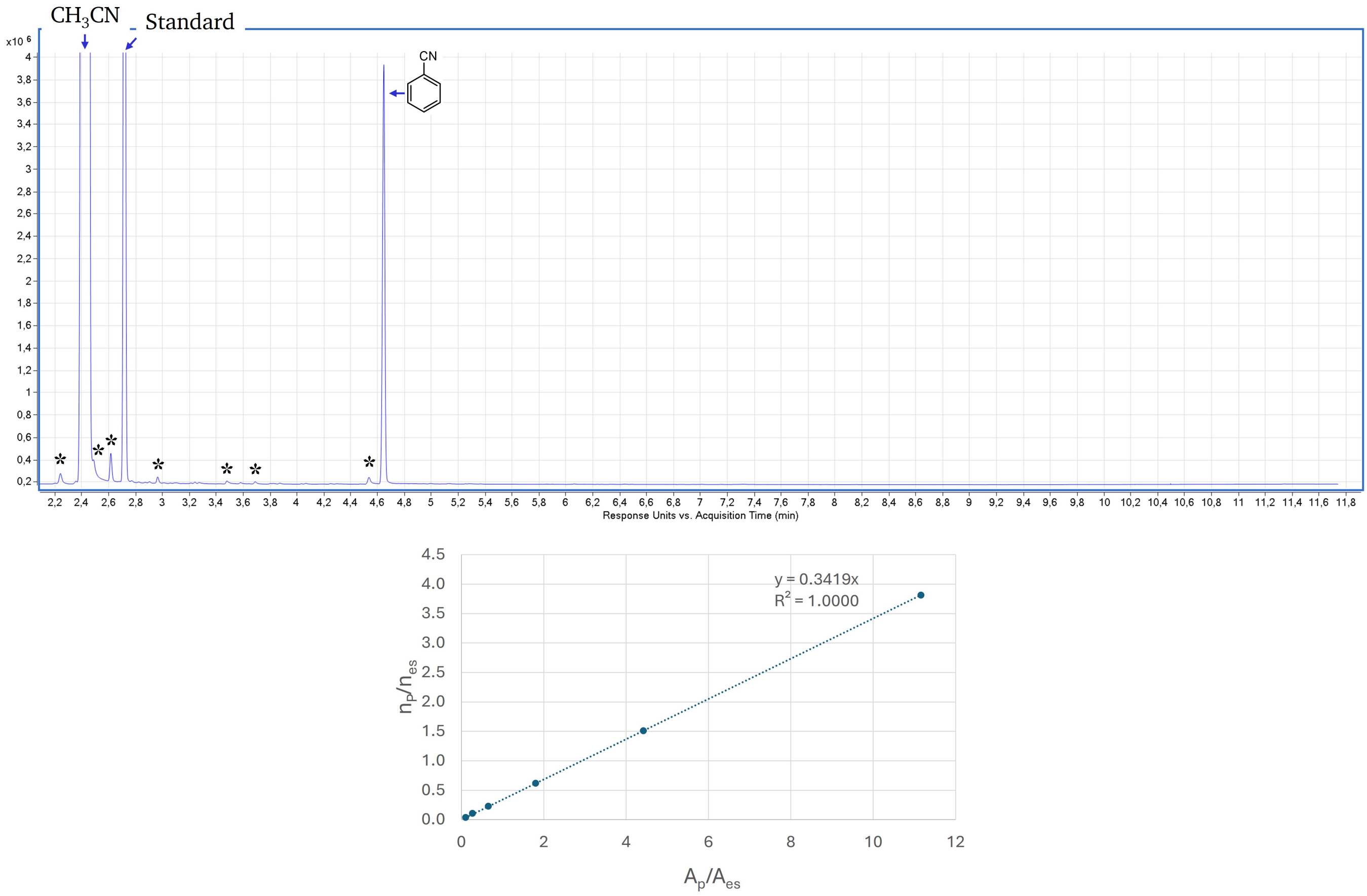

Fig. SI. 13 GC-FID chromatogram of the liquid phase collected at the microreactor outlet after exposure of a benzonitrile in acetonitrile solution to an argon plasma (10 kV_{pp}, 1.5 kHz, 10 °C , 8 10^{-2} mol/L, Q_g = 0.05 mL/min, Q_l = 0.01 mL/min). Products deriving from the reactivity of neat acetonitrile are indicated with a star. The calibration curve of benzonitrile using ethyl acetate as the external standard is also provided.

9.2 Cyanation of hexafluorobenzene

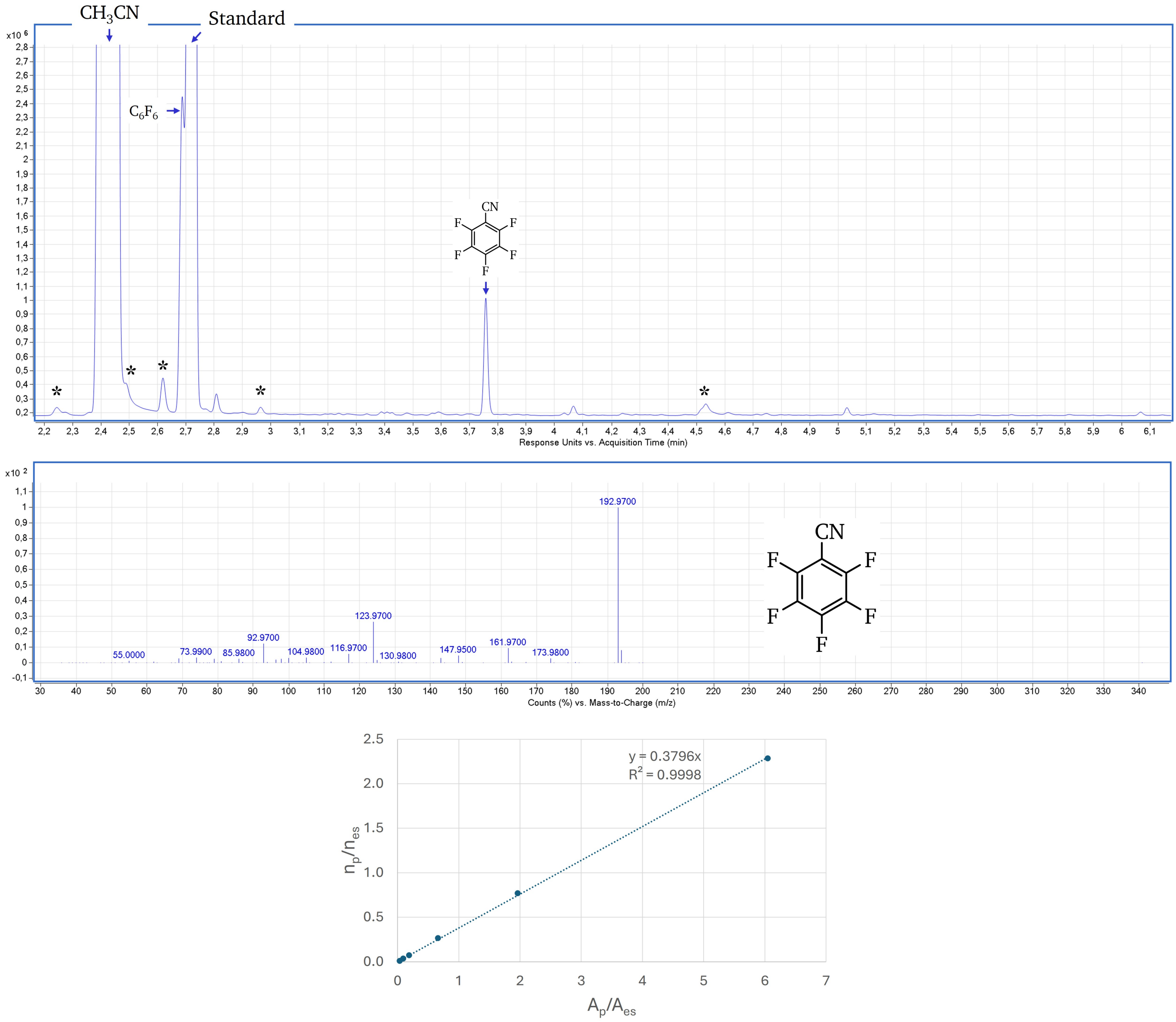

Fig. SI. 14 GC-FID chromatogram of the liquid phase collected at the microreactor outlet after exposure of a hexafluorobenzene in acetonitrile solution to an argon plasma (10 kV$_{pp}$, 1.5 kHz, 10 °C , 8 10^{-2} mol/L, Q_g = 0.05 mL/min, Q_l = 0.01 mL/min). Products deriving from the reactivity of neat acetonitrile are indicated with a star. The calibration curve of hexafluorobenzene using ethyl acetate as the external standard and the mass spectrum of the main product is also provided.

9.3 Cyanation of fluorobenzene

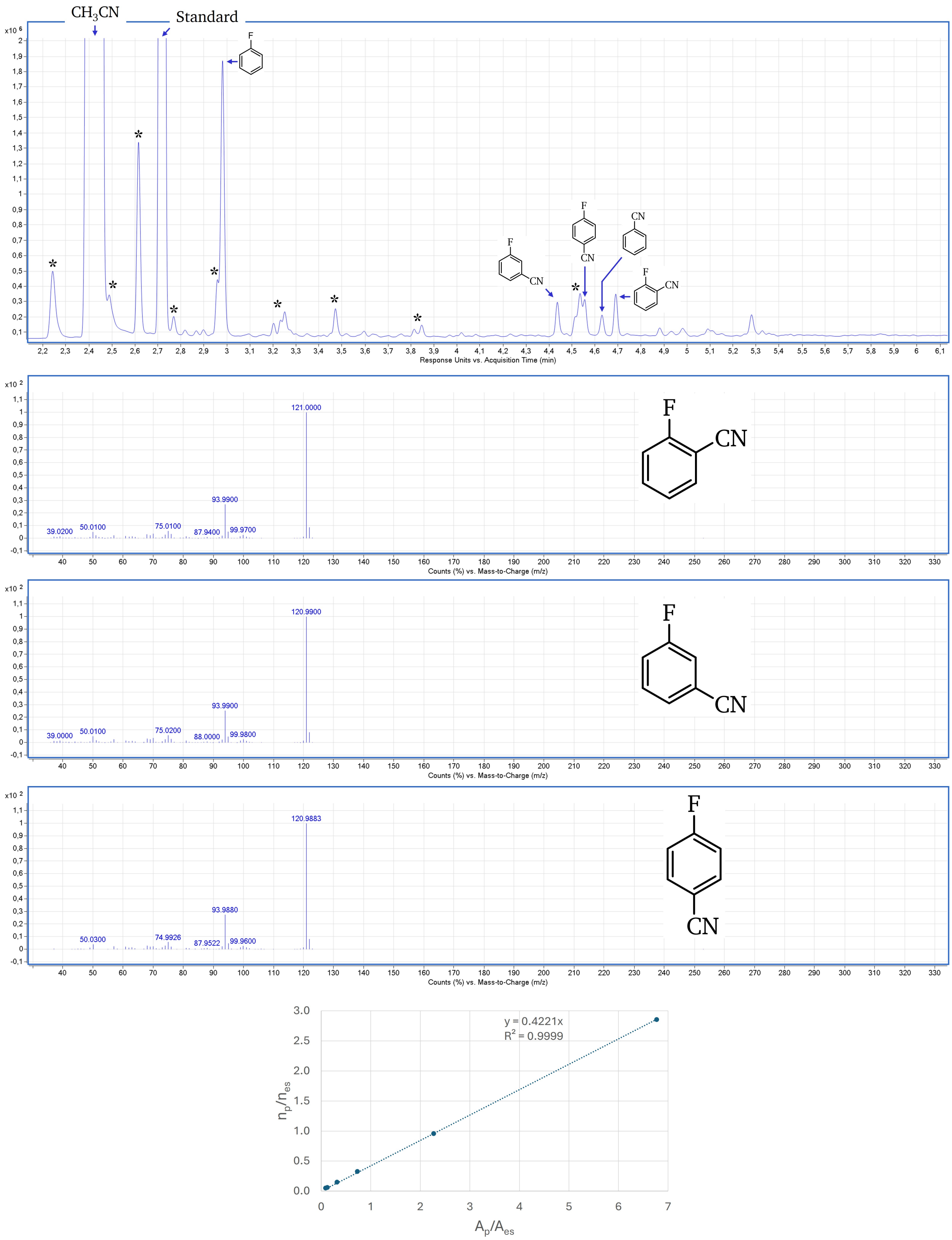

Fig. SI. 15 GC-FID chromatogram of the liquid phase collected at the microreactor outlet after exposure of a fluorobenzene in acetonitrile solution to an argon plasma (10 kV_{pp}, 1.5 kHz, 10 °C , 8 10^{-2} mol/L, Q_g = 0.05 mL/min, Q_l = 0.01 mL/min). Products deriving from the reactivity of neat acetonitrile are indicated with a star. The calibration curve of fluorobenzene using ethyl acetate as the external standard and the mass spectra of the main products are also provided.

9.4 Cyanation of chlorobenzene

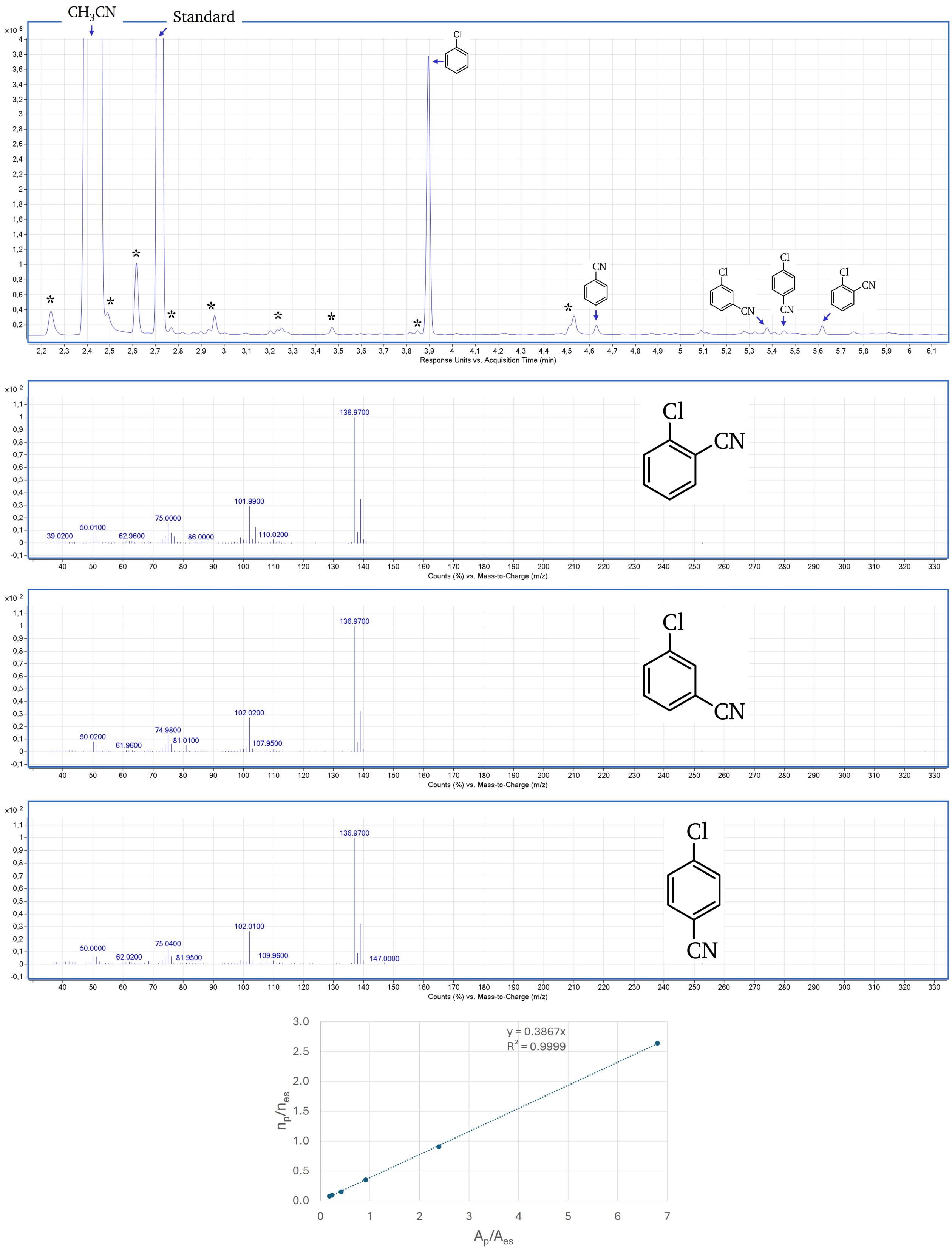

Fig. SI. 16 GC-FID chromatogram of the liquid phase collected at the microreactor outlet after exposure of a chlorobenzene in acetonitrile solution to an argon plasma (10 kV_{pp}, 1.5 kHz, 10 °C , 8 10^{-2} mol/L, Q_g = 0.05 mL/min, Q_l = 0.01 mL/min). Products deriving from the reactivity of neat acetonitrile are indicated with a star. The calibration curve of chlorobenzene using ethyl acetate as the external standard and the mass spectra of the main products are also provided.

9.5 Cyanation of toluene

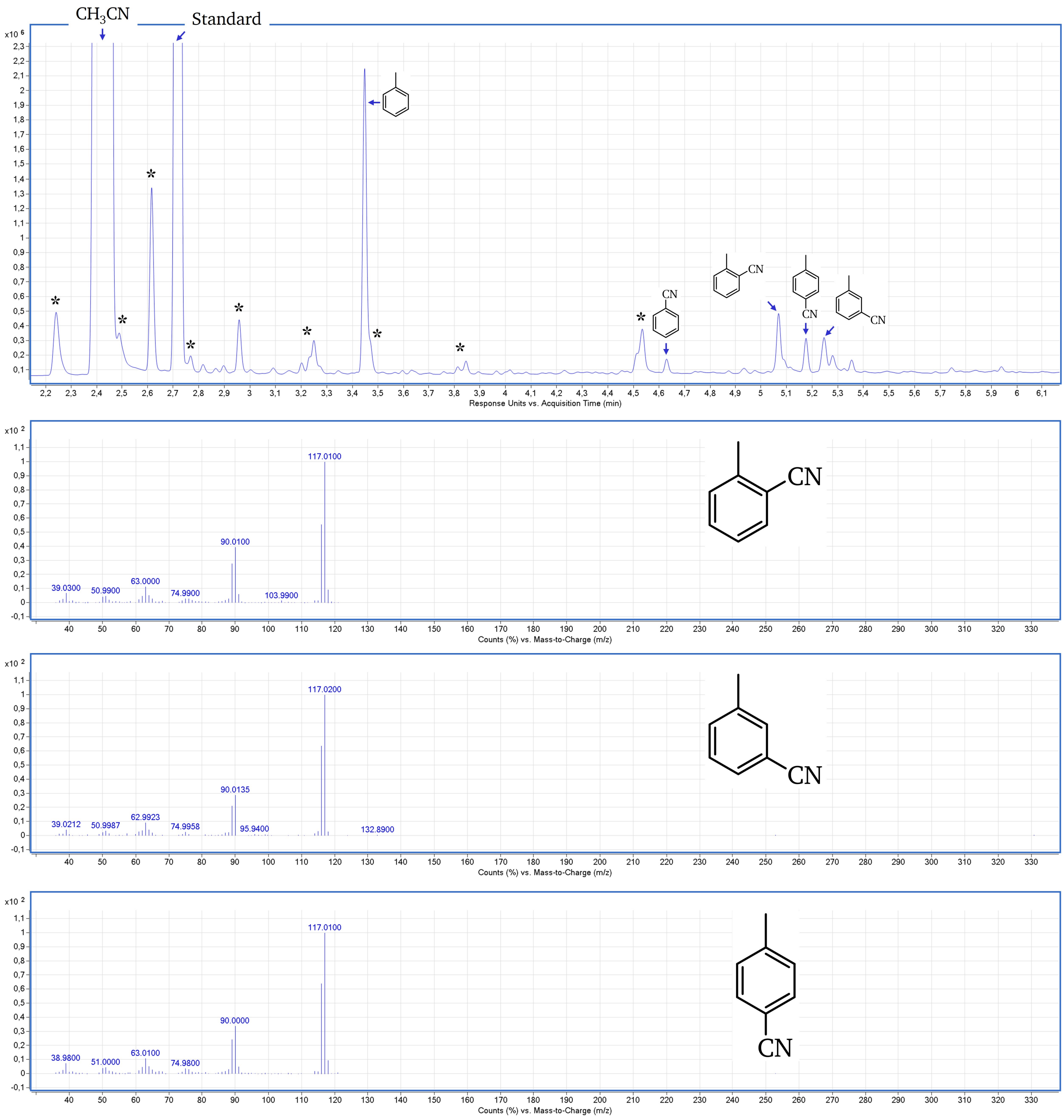

Fig. SI. 17 GC-FID chromatogram of the liquid phase collected at the microreactor outlet after exposure of a toluene in acetonitrile solution to an argon plasma (10 kV_{pp}, 1.5 kHz, 10 °C , 8 10^{-2} mol/L, Q_g = 0.05 mL/min, Q_l = 0.01 mL/min). Products deriving from the reactivity of neat acetonitrile are indicated with a star. The mass spectra of the main products are also provided. Toluene response factor using ethyl acetate as the external standard was extracted from the literature.[6]

9.6 Cyanation of ethylbenzene

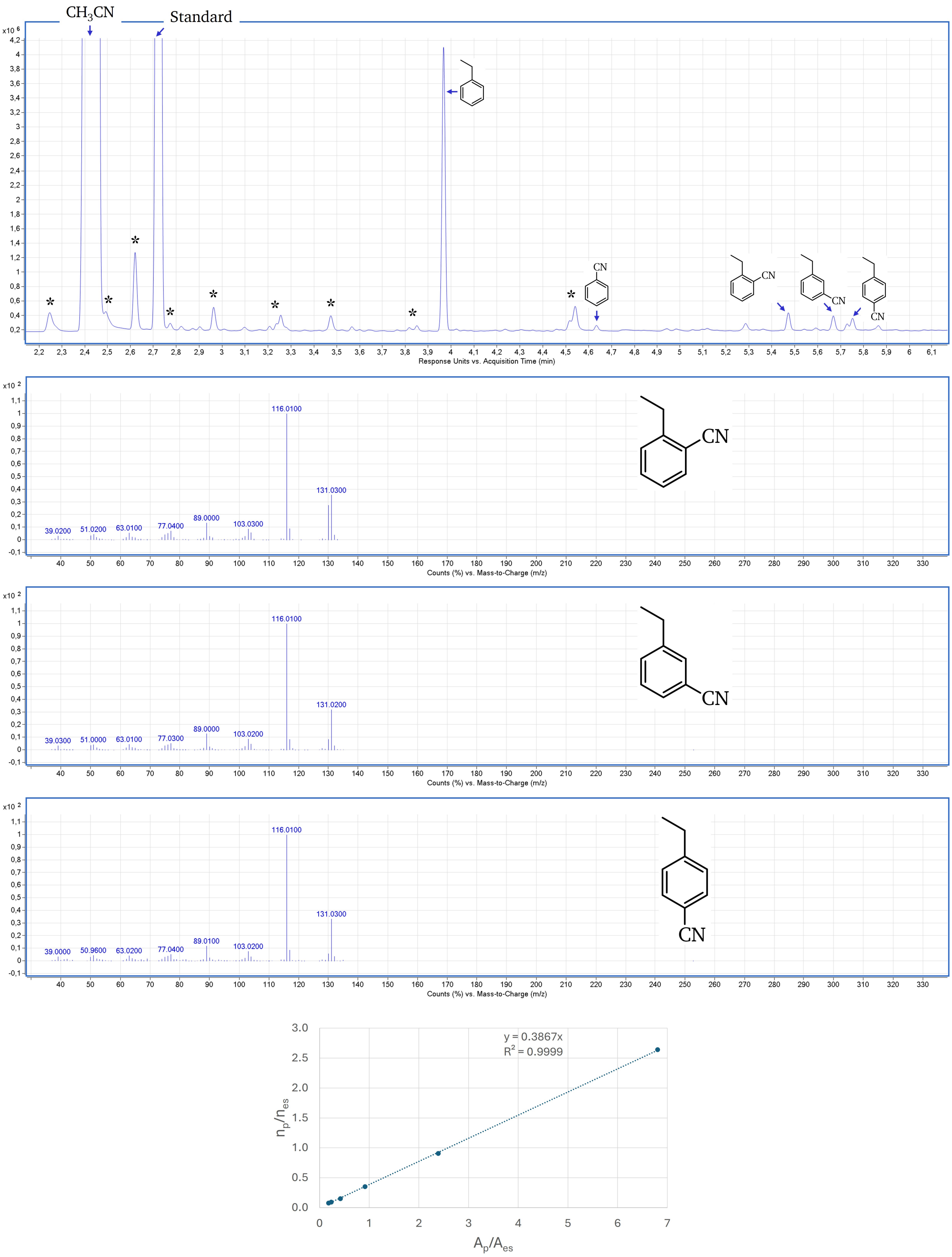

Fig. SI. 18 GC-FID chromatogram of the liquid phase collected at the microreactor outlet after exposure of an ethylbenzene in acetonitrile solution to an argon plasma (10 kV_{pp}, 1.5 kHz, 10 °C , 8 10^{-2} mol/L, Q_g = 0.05 mL/min, Q_l = 0.01 mL/min). Products deriving from the reactivity of neat acetonitrile are indicated with a star. The calibration curve of ethylbenzene using ethyl acetate as the external standard and the mass spectra of the main products are also provided.

9.7 Cyanation of trifluoromethoxybenzene

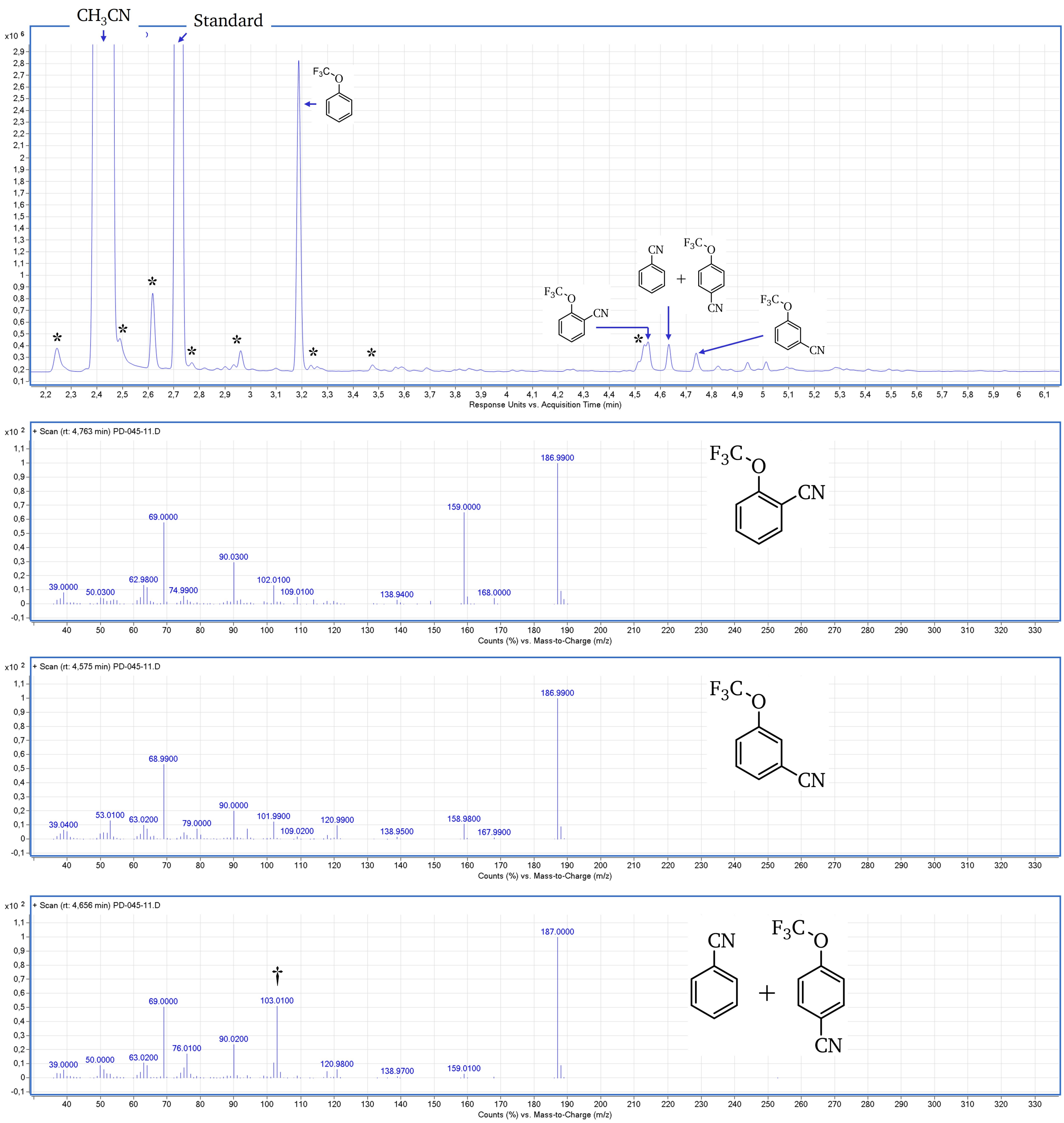

Fig. SI. 19 GC-FID chromatogram of the liquid phase collected at the microreactor outlet after exposure of a trifluoromethoxybenzene in acetonitrile solution to an argon plasma (10 kV_{pp}, 1.5 kHz, 10 °C , 8 10^{-2} mol/L, $Q_g = 0.05$ mL/min, $Q_l = 0.01$ mL/min). Products deriving from the reactivity of neat acetonitrile are indicated with a star. The mass spectra of the main products are also provided. The † symbol indicates the caracteristic peak of benzonitrile, which was used to calculate benzonitrile and p-trifluoromethoxybenzonitrile yields separately.

9.8 Cyanation of anisole

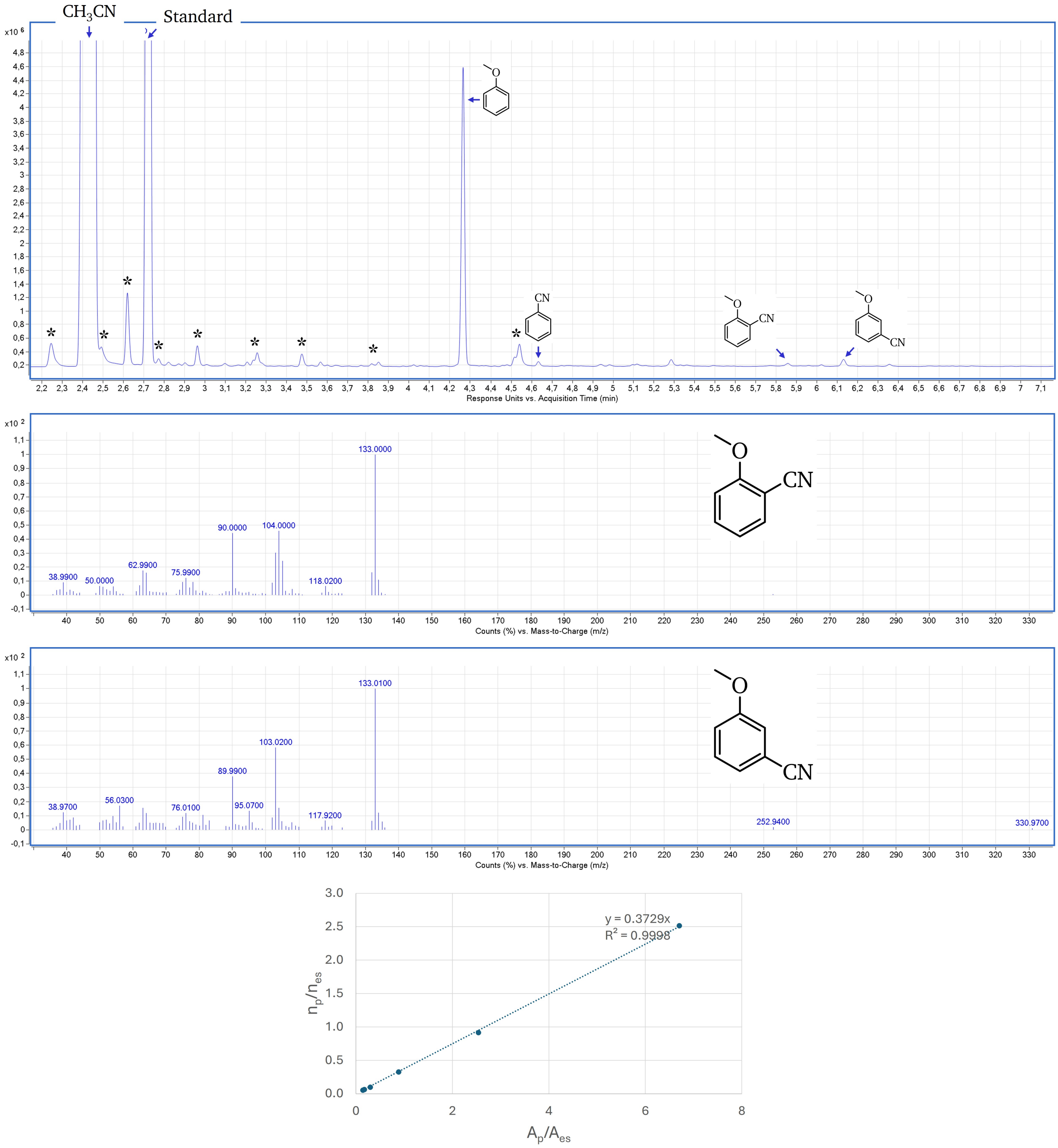

Fig. SI. 20 GC-FID chromatogram of the liquid phase collected at the microreactor outlet after exposure of an anisole in acetonitrile solution to an argon plasma (10 kV_{pp}, 1.5 kHz, 10 °C , 8 10^{-2} mol/L, Q_g = 0.05 mL/min, Q_l = 0.01 mL/min). Products deriving from the reactivity of neat acetonitrile are indicated with a star. The calibration curve of anisole using ethyl acetate as the external standard and the mass spectra of the main products are also provided.

References

1 Holub, M. On the measurement of plasma power in atmospheric pressure DBD plasma reactors. *International Journal of Applied Electromagnetics and Mechanics* **2012**, *39*, 81–87.

2 Dedieu, P.; Morand, G.; Loubière, K.; Ognier, S.; Tatoulian, M. Microreactor designed for efficient plasma–liquid segmented flows. *Lab on a Chip* **2024**, *24*, 3898–3908.

3 *NIST chemistry WebBook, NIST Standard Reference Database Number 69*, p.j. linstrom and w.g. mallard ed.; 2004.

4 Lepoetre, A.; Ognier, S.; Zhang, M.; Wengler, J.; Al Ayoubi, S.; Ollivier, C.; Fensterbank, L.; Duten, X.; Tatoulian, M. Amination of Cyclohexane by Dielectric Barrier Discharge Processing in a Continuous Flow Microreactor: Experimental and Simulation Studies. *Plasma Chemistry and Plasma Processing* **2021**, *41*, 351–368.

5 Zhang, M.; Ognier, S.; Touati, N.; Hauner, I.; Guyon, C.; Binet, L.; Tatoulian, M. A plasma/liquid microreactor for radical reaction chemistry: An experimental and numerical investigation by EPR spin trapping. *Plasma Processes and Polymers* **2018**, *15*, 1700188.

6 Bruel, F.; Morand, G.; Ognier, S.; Dedieu, P.; Favre-Réguillon, A.; Ollivier, C.; Fensterbank, L.; Tatoulian, M. Direct C(sp^3)–H chloromethylation through CH_2Cl_2 activation using non-thermal plasma. *Green Chemistry* **2025**, *27*, 11857–11869.
